# Molecular diagnose of a large hearing loss population from China by targeted genome sequencing

**DOI:** 10.1038/s10038-022-01066-5

**Published:** 2022-08-19

**Authors:** Jie Wu, Zongfu Cao, Yu Su, Yang Wang, Ruikun Cai, Jiyue Chen, Bo Gao, Mingyu Han, Xiaohong Li, DeJun Zhang, Xue Gao, Shasha Huang, Quanfei Huang, Yongyi Yuan, Xu Ma, Pu Dai

**Affiliations:** 1 Key Lab of Hearing Impairment Science of Ministry of Education, Key Lab of Hearing Impairment Prevention and Treatment of Beijing, National Clinical Research Center for Otolaryngologic Diseases, College of Otolaryngology Head and Neck Surgery, Chinese PLA General Hospital, Chinese PLA Medical School, #28 Fuxing Road, Beijing, 100853 China; 2 National Research Institute for Family Planning, National Human Genetic Resource Center, No. #12 Dahuisi Road, Beijing, 100081 China; 3 Capital Bio Genomics Co. Dongguan, No. #1 Taoyuan Road, Dongguan City, Guangdong, 523808 China; 4 Department of Otolaryngology Head and Neck Surgery, Beijing Children’s Hospital, National Center for children’s Health, #56 South Lishi Road, Xicheng District, Beijing, 100045 China; 5 Department of Otolaryngology Head and Neck Surgery, The Second Hospital of Jilin University, #218 Ziqiang Street, Nanguan District, Changchun City, Jilin, 130041 China; 6 Department of Otolaryngology, PLA Rocket Force Characteristic Medical Center, #16 XinWai Da Jie, Beijing, 100088 China

**Keywords:** Genetic testing, Next-generation sequencing

## Abstract

Hereditary hearing loss is genetically heterogeneous, with diverse clinical manifestations. Here we performed targeted genome sequencing of 227 hearing loss related genes in 1027 patients with bilateral hearing loss and 520 healthy volunteers with normal hearing to comprehensively identify the molecular etiology of hereditary hearing loss in a large cohort from China. We obtained a diagnostic rate of 57.25% (588/1027) for the patients, while 4.67% (48/1027) of the patients were identified with uncertain diagnoses. Of the implicated 35 hearing loss genes, three common genes, including *SLC26A4*(278/588), *GJB2*(207/588), *MT-RNR1*(19/588), accounted for 85.54% (503/588) of the diagnosed cases, while 32 uncommon hearing loss genes, including *MYO15A*, *MITF*, *OTOF*, *POU3F4*, *PTPN11*, etc. accounted for the remaining diagnostic rate of 14.46% (85/588). Apart from Pendred syndrome, other eight types of syndromic hearing loss were also identified. Of the 64 uncertain significant variants and 244 pathogenic/likely pathogenic variants identified in the patients, 129 novel variants were also detected. Thus, the molecular etiology presented with high heterogeneity with the leading causes to be *SLC26A4* and *GJB2* genes in the Chinese hearing loss population. It’s urgent to develop a database of the ethnicity-matched healthy population as well as to perform functional studies for further classification of uncertain significant variants.

## Introduction

More than 0.5 billion people are known to be affected by hearing loss (HL) worldwide [1], and the figure is expected to reach approximately 2.5 billion by 2050 (https://www.who.int). This medical condition is known to cause various adverse effects in the affected individuals [2–4]. The etiology of HL involves genetic causes, nongenetic causes, and a combination of these two factors [5, 6]. It has been estimated that about 60% of the patients with HL have hereditary hearing loss (HHL) [7], and the genetic causes vary dramatically across different ethnic populations globally [8, 9].

HHL is highly heterogeneous, both in genotype and phenotype. Until now, more than 140 HL genes have been identified, and the inheritance patterns of these genes involve autosomal recessive (AR), autosomal dominant (AD), X-linkage, and mitochondrial inheritance (http://hereditaryhearingloss.org). Thus, the clinical manifestations of HHL are diverse. The various types of HL include sensorineural, conductive, and mixed HL, while the severity of HL includes mild, moderate, severe, and profound, and can occur at any age of life. Apart from simple HL, the genetic causes can also lead to syndromic hearing loss [6]. Additionally, apart from monogenetic inheritance, digenetic inheritance in HL patients was also reported [10–12]. Simultaneously, there was still reports that didn’t support the digenetic inheritance pattern in HL [13, 14].

Given the large number of HL genes, the advent and development of high-throughput sequencing technology has revolutionized the identification of molecular etiology of HHL [15, 16]. The massively parallel sequencing has become an efficient routine diagnosis and research method in this field [17]. Subsequently, more patients have been found to obtain positive diagnoses related to HL genes apart from the common HL genes, such as *GJB2*, *SLC26A4*, and *MT-RNR1* in the Chinese population [18, 19].

In the present study, we focused on monogenic inheritance in HL and aimed to assess the contribution of genetic factors in HL in a large cohort from China and identify the gene spectrum in this cohort. We enrolled 1577 subjects from China, including 1027 patients with bilateral HL and 520 healthy volunteers with normal hearing and tested them using targeted genome enrichment and multiple parallel sequencing for the 277 HL-related genes. These results would enhance our understanding of the molecular etiology of HL in the Chinese population to help guide the medical care and facilitate genetic counseling to the patients and their family members [20].

## Materials and methods

### Subjects

The Ethics Committee of Chinese PLA General Hospital approved this study (No. S2016-120-01), which was performed consistently with the Declaration of Helsinki. For all participants or the parents of the minors, written informed consent was obtained.

In this study, besides 520 healthy volunteers with normal hearing, 1027 unrelated probands with bilateral hearing loss were also enrolled who had been referred to the genetic testing center for deafness during the period of 2015–2017 and were all tested for common HL genes, including *GJB2*, *SLC26A4*, and *MT-RNR1*(m.A1555G, m.C1494T) by Sanger sequencing.

The audiological evaluation was performed by pure tone audiometry. For those subjects who could not undergo pure tone audiometry, auditory steady-state response or behavior auditory testing or auditory brainstem response were measured. Hearing levels were determined by the average threshold at the frequency of 0.5, 1, 2, and 4 KHz of the better ear for pure tone audiometry, auditory steady-state response, and behavior auditory testing, or response threshold for auditory brainstem response. Other audiometric testing techniques, including otoacoustic emissions, 40 Hz auditory event-related potentials (40 Hz AERP), etc. were recommended if required. The severity of HL was graded as follows: mild (26–40 dB), moderate (41–55 dB), moderately severe (56–70 dB), severe (71–90 dB), and profound (>90 dB) [21]. Asymmetric hearing was defined as the difference in the mean level at the frequency of 0.5, 1, 2, and 4 KHz or three contiguous frequencies between two ears bigger than 15 dB [22–24].

High-resolution computed tomography of the temporal bones was performed to evaluate malformations of the inner ear structure. For patients with syndromic HL, other physical examinations were recommended if required.

Physical examination was performed for healthy volunteers, including testing of body temperature, height, body weight, pulse, blood pressure, Electrocardiogram, transabdominal ultrasound, chest X-ray, psychiatric examination, neurologic examination, otolaryngological examination, optical examination. The hearing level determined by pure tone audiometry was smaller than 25 dB for both ears.

DNA was extracted from peripheral leukocytes of each subject and the family members using standard protocol.

### Sequencing

The following 227 HL-related genes were included in this study: 60 genes related to AR non-syndromic HL, 27 genes related to AD non-syndromic HL, 5 genes related to X-link HL, 34 genes related to syndromic HL, and other 101 genes related to genetic disease with HL phenotype recorded in Mendelian Inheritance in Man.

Agilent SureDesign online tool (https://erray.chem.agilent.com/suredesign/) was used to design the probes targeting all the exons, flanking intronic sequences (±10 bp), and known pathogenic variants located in introns of the 227 HL-related genes. Thus, 4544 regions encompassing 1.101 Mbp of the genome were targeted. Ion Plus Fragment Library Kit (Agilent Technologies, Santa Clara, CA) was used for library preparation, with the DNA fragments approximately 170 bp long. SureDesign hybridization capture technology (Agilent Technologies, Santa Clara, CA) was applied following the instruction of the manufacturer. The prepared DNA samples were subjected to JingXin BioelectronSeq 4000 System semiconductor sequencer (CFDA registration permit NO. 20153400309).

### Bioinformatics analysis

Torrent Suite Software v5.4 (Thermo Fisher Scientific, Waltham, MA) analysis pipeline was used to produce high-quality read files. After quantity control, the sequence reads were aligned to the human reference sequence genome (hg19) by Torrent Mapping Alignment Program (3.6.40). Picard (1.84) was used to remove the repeated reads. Torrent Variant Caller software v5.4–11 was used to detect the single nucleotide variants (SNVs) and insertion and deletion (INDEL) variation.

### Variant interpretation

The detected variants with read depth < 5X were filtered out. ANNOVAR (20170601) was used to annotate the variants. Variants with minor allele frequency >0.05 as reported in the population database, including dbSNP (http://www.ncbi.nlm.nih.gov/snp)(20170929), 1000 Genome Project (http://www.browser.1000genomes.org)(20150824), and Genome Aggregation Database (gnomAD; http://gnomad.broadinstitute.org)(20170311) were filtered out. We used SIFT (20170221), PolyPhen-2(20170221), MCAP (20170221), REVEL (20161205), MutationTaster (20170221), PROVEAN (20170221), to predict the damage of the variants.

The detected candidate variants were further interpreted by considering the allelic frequency in the control group of 520 individuals with normal hearing and referring to the database of Deafness Variation Database (2020-07-30) (http://deafnessvariationdatabase.org), ClinVar (2020-07-30) (http://www.ncbi.nlm.nih.gov/clinvar) as well as our internal database. Novel variants were determined as that hadn’t been previously reported in databases including ClinVar and dbSNP. The identified novel variants in this study were submitted to the CinVar database. Furthermore, the correlation between candidate variants and the phenotype of the affected individuals were considered on a patient-by-patient basis. Variants were confirmed by Sanger sequencing in the families. For de novo variants, the paternity and maternity were verified by genotype analysis by short tandem repeat typing assay. Finally, the variants were classified according to the ACMG guidelines and the specification of guidelines for HHL [25, 26].

### Splicing assay

For some detected splice variants, minigene assay was performed to validate the impact on splicing [27, 28]. The pair of minigene clones, which carried wild-type sequence or variant sequence of interest, were transfected into HEK-293T cells, respectively.

### Statistical analysis

Chi-squared analyses were performed to compare the difference among groups using SPSS Statistics 25. The statistical significance was defined as *P* < 0.05.

## Results

### Targeted capture sequencing

We tested 1547 subjects using targeted genome sequencing. An average of 99%, 98.7%, 98%, 97% of the targeted bases for the 227 genes related to HL (Table S1) was covered at 1X, 5X, 10X, 20X reads, respectively.

### Genetic diagnosis

Table 1 presents the clinical information of the patients. Healthy volunteers included 352 male and 168 female subjects, aged 18 to 58 years, with an average of 30.79 ± 9.15 years.

**Table 1:** Clinical information of 1027 patients included in this study

Characteristic	Number	Percentage
Gender		
Male	554	53.94%
Female	473	46.06%
Age of onset/awareness(years)		
0	403	39.24%
>0 and ≤2	349	33.98%
>2 and ≤5	186	18.11%
>5 and ≤9	42	4.09%
>9 and ≤19	31	3.02%
>19	3	0.29%
NP	13	1.27%
Nationality		
Han	972	94.64%
Dai	1	0.10%
Manchu	19	1.85%
Gelao	1	0.10%
Mongolian	9	0.88%
Hui	10	0.97%
Tujia	9	0.88%
Daur	1	0.10%
Bouyei	1	0.10%
Miao	1	0.10%
Korean	1	0.10%
NP	2	0.19%
Geographical location		
Northeast	67	6.52%
North	339	33.01%
Central	144	14.02%
East	391	38.07%
South	15	1.46%
Northwest	42	4.09%
Southwest	25	2.43%
NP	4	0.39%
Family history		
No	893	86.95%
Yes	126	12.27%
NP	8	0.78%
Severity		
Mild	18	1.75%
Moderate	75	7.30%
Moderate severe	155	15.09%
Severe	196	19.08%
Profound	550	53.55%
NP	33	3.21%
Symmetry		
Symmetric	777	75.66%
Asymmetric	168	16.36%
NP	82	7.98%
Syndrome		
Non-syndromic	668	65.04%
Syndromic	317	30.87%
NP	42	4.09%

*NP* not provided

**Fig. 1:**
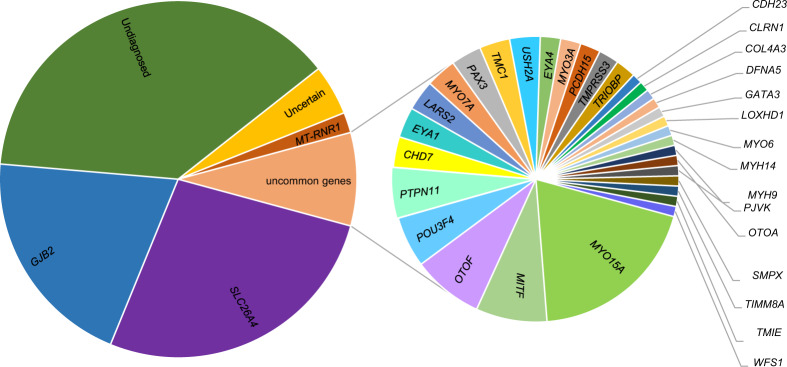
The molecular diagnostic yields of the 1027 HL patients from China in this study

Of the 1027 HL patients, the genetic cause was identified in 588 patients as variants of pathogenic or likely pathogenic were considered. Other 48 patients (uncertain significance variant in *POU4F3* and causative variant in *GJB2* were simultaneously detected in one case) were identified as uncertain in whom at least one uncertain significance variant (VUS) was identified in one allele in HL genes, even if a pathogenic/likely pathogenic variant was detected in another allele in the AR genes. The remaining 392 patients were categorized as undiagnosed (Fig. 1).

Additionally, 35 HL genes were implicated in the diagnosed patients, and two leading genes were *SLC26A4* (278/588) and *GJB2* (207/588), as previously reported [29]. The causative variants in *MT-RNR1* were detected in 19 patients (18 cases with m.A155G and one case with m.C1494T). These three genes were considered as common HL genes in China [19, 29].

Then, 32 uncommon genes accounted for the remaining 86 diagnosed patients (causative variants in *SLC26A4* and *COL3A4* were simultaneously identified in one patient). Genes that were detected in more than three patients included *MYO15A* (17/86), *MITF* (7/86), *OTOF* (7/86), *POU3F4* (5/86), *PTPN11* (5/86), *TMC1* (3/86), *LARS2* (3/86), *PAX3* (3/86), *EYA1* (3/86), *CHD7* (3/86). *MYO7A* (3/86) and *USH2A* (3/86). In this patient subgroup of 86 cases, 55 were diagnosed as non-syndromic HL. The remaining 27 patients were identified to be syndromic HL (Table 2). The other four patients with variants in *USH2A*, *CLRN1*, the responsible genes for Usher syndrome, in whom the ophthalmic phenotype was not observed, were classified as non-syndromic HL (NSHL) mimics [30].

**Table 2:** The syndromic HL detected in this study

Syndrome	Gene	Patient number
Pendred Syndrome	*SLC26A4*	235
CHARGE Syndrome	*CHD7*	3
Alport Syndrome	*COL4A3*	1
Branchio-Oto-Renal Syndrome	*EYA1*	3
Barakat Syndrome	*GATA3*	1
Perrault Syndrome	*LARS2*	3
Waardenburg Syndrome	*MITF*	7
	*PAX3*	3
LEOPARD/Noonan Syndrome	*PTPN11*	5
Deafness-Dystonia-Optic Neuronopathy Syndrome	*TIMM8A*	1

### Variant identification

We identified 20 pathogenic/likely pathogenic variants in *GJB2* (Table S2) as the genetic cause in 207 patients. The two leading causative variants were NM_004004.6: c.235delC and c.299-300del, which were detected in 84.06% (174/207) and 32.37% (67/207) of this patient subgroups, respectively.

Next, 85 pathogenic/likely pathogenic variants in *SLC26A4* (Table S3) were identified as the underlying molecular etiology of 278 patients diagnosed as Pendred syndrome or simple HL with enlarged vestibular aqueduct, of which 26 variants had not been previously reported. The most common two causative variants of *SLC26A4* were NM_000441.2: c.919-2 A > G and c.2168 G > A, which were detected in 21.22% (59/278) and 76.26% (212/278) of the *SLC26A4* related patients, respectively. Table S4 presents the phenotype information of patients caused by variants in *GJB2* and *SLC26A4*.

We also identified 117 pathogenic/likely pathogenic variants in 32 uncommon HL genes as the molecular causes in 86 patients (Table S5), of which, 19 de novo variants in AD or X-linked HL genes were detected.

Additionally, we identified 64 VUS in 24 HL genes in 48 patients, and these variants were all point variants. Additionally, 20 pathogenic/likely pathogenic variants were also identified in this patient subgroup (Table S6).

### Validation of two splice variants

**Fig. 2:**
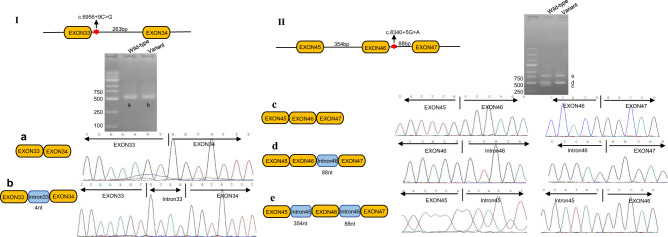
The impact of two splice variants in *MYO15A* gene on the splicing pattern. Wild-type: the amplification samples from cells transfected with plasmid carrying wild-type sequence of interest; Variant: the samples from transfectants carrying variant sequence of interest; I: The variant of c.6956 + 9 C > G trapped 4 nucleotides(nt) of intron33 of *MYO15A* gene; II: The variant of c.8340 + 5 G > A trapped intron45 and intron46 of *MYO15A* gene

The results of minigene assay showed NM_016239.4: c.6956 + 9 C > G in *MYO15A* trapped 4 nucleotides(nt) of intron33 while c.8340 + 5 G > A trapped the intron45 and intron46, which indicated these two splice variants altered the expression pattern of this gene (Fig. 2).

### Phenotypes and diagnostic rate

**Fig. 3:**
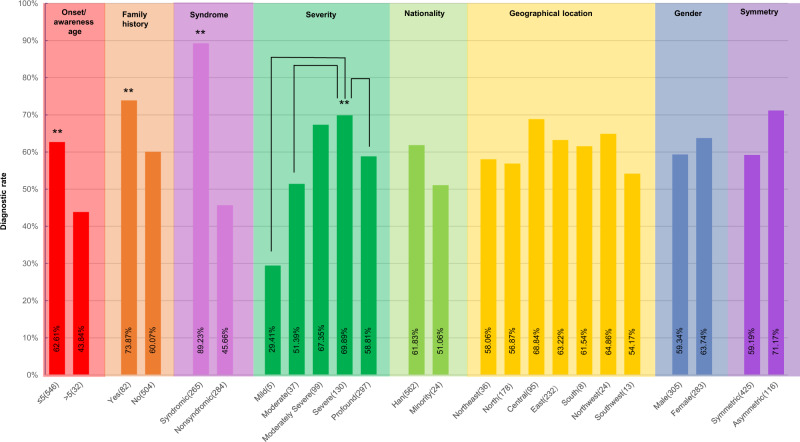
The impact of phenotypes on the diagnostic rate. The diagnostic rate(%) = the number of diagnosed patients subgroup/the summary of diagnosed and undiagnosed patients subgroup × 100%. The statistical significance was defined as **P < 0.01

The impact of clinical phenotypes on diagnostic rate were analyzed, including gender, onset/awareness age, family history, the severity of HL, the symmetry of the two affected ears, geographical location, and nationality (Fig. 3, Table S7). Compared with that of the probands without family history, the diagnostic rate of the probands with family history was significantly higher (73.87% vs. 60.07%, *P* < 0.01). Similarly, the diagnostic rate of the patients with the onset/awareness age below five years was 62.69% (*P* < 0.01), and that of the patients with syndromic HL was 89.23% (*P* < 0.005). This result indicated that the genetic cause played a significant role in the etiology of these three subgroups.

The diagnostic rate of patients with mild HL (29.41%) or with moderate HL (51.39%) or with profound HL (58.51%) was significantly lower than that of patients with severe HL (69.89%) (*P* < 0.005, *P* < 0.005, *P* < 0.01, respectively). This difference might have arisen from the patient subgroup caused by variations in *SLC26A4*: if we excluded the patients related to *SLC26A4* from the diagnosed group, the diagnosis rate wouldn’t be significantly different among all the subgroups with different HL levels (*P* > 0.05).

Of the 577 patients with High-resolution computed tomography imaging available (excluding the patients with enlarged vestibular aqueduct and incomplete partition type III, 297 cases altogether, which was highly correlated to *SLC26A4* and *POU3F4*, respectively), 31 cases were diagnosed with inner ear malformation (Fig. S1) [31]. Of these 31 patients, 28 did not obtain molecular diagnoses (Table S8), while only three patients with cochlear hypoplasia IV type were identified to be related to the *EYA1* gene. This result indicated that there was a necessity to further study the etiology of this molecularly undiagnosed inner ear malformations [32, 33].

## Discussion

### The case-control study

The allele frequency of variants in an ethnicity-matched healthy population is very useful for the classification of variants [25, 26]. Herein, to explore the molecular etiology of a large cohort from China, a case-control study was performed. For the detected variants, apart from the allele frequency in the publicly available population databases, the frequency of variants detected in this control group was also considered (Tables S2, S3, S5, S6). For example, NM_206933.4:c.8559-2 A > G in *USH2A*, the allele frequency in the control group was 4/2054, while that in the patient group was 4/1040. Based on the data in this study, this variant was identified as VUS while it was classified as pathogenic in the Deafness Variation Database. However, we still noted that the number of the control group was relatively less compared with that of the patient group, which implied the urgency of the setup of the HL variants database of the ethnicity-matched healthy population to improve variant interpretation.

For the patient group, there were no exclusive criteria except the bilateral HL, which was supposed to be more likely related to hereditary etiology than the unilateral HL [30]. Furthermore, the patients were all pre-screened for common HL genes, including *GJB2*, *SLC26A4*, and *MT-RNR1*(m.A1555G, m.C1494T) by Sanger sequencing. Thus, the diagnoses of HL caused by *MT-RNR1* were obtained from this first-generation sequencing.

### The diagnosis of the patients

In this study, three common genes, including *GJB2*, *SLC26A4*, *MT-RNR1*, accounted for 85.54% (503/588, 35.20%, 47.28%, 3.23%, respectively) of the diagnostic patient group, while 32 uncommon HL-related genes accounted for the remaining 14.46% (85/588) of the diagnostic yield. While it has been reported in 459 HL patients from the United States, 28% (128) had positive genetic testing, with the leading five involved genes as *GJB2*, *TMPRSS3*, *SLC26A4*, *MYO7A*, and *MT-RNR1* (16%, 10%, 8%, 7%, 5%, respectively) [34]. Another report presented that 56% of 2198 HL patients from 491 Palestinian families was genetic, and the top five genes implicated were *GJB2*, *MYO15A*, *SLC26A4*, *MYO7A*, and *CDH23* (22%, 11%, 8.9%, 8.3%, 5%, respectively) with most common variant to be c.35delG in *GJB2*, c.1001 G > T in *SLC26A4*, and c.7207 G > T in *MYO15A* [35]. Though the including criteria for the subjects were different form each other in these published reports, we still could speculate that the spectrum and frequency of the molecular etiology in this Chinese patient cohort was different from that of other populations [36–38].

Here, we focused on sequence variants mostly located in the exons of 227 HL-related genes. In addition, there were other causative variants that were not covered in this panel, including (1) variants located in other non-coding regions of the targeted genes; (2) other variant types, for example, copy number variant, which has been testified to be involved in the HL; (3) unknown novel HL genes [39, 40]. If the above-mentioned variant types that this panel did not cover and 48 uncertain diagnosed patients were considered, the proportion of HHL in this patient cohort would be greater than 57.25% (588/1027).

### Impact of phenotypes on the diagnostic rate

In the analysis of clinical phenotypes on the molecular diagnostic rate, 588 diagnosed patients and 370 undiagnosed patients were included, the diagnostic rate(%) = the number of diagnosed patients of subgroup/the summary of diagnosed and undiagnosed patients of subgroup × 100% (Table S7). In the calculation of diagnostic rate, 22 undiagnosed patients with characteristic phenotypes which are highly correlated to the HHL were excluded from the undiagnosed patient group (392), including 18 patients with enlarged vestibular aqueduct carrying one pathogenic variant in *SLC26A4*, three patients diagnosed as Waardenburg syndrome, and one case with inner malformation of IP-III. Therefore, there was 370 undiagnosed patients were taken into account in the analysis of the diagnostic rate.

In the current study, we noticed that the affected individuals from minority nationalities only comprised 5.16% of the patient group. Similarly, probands from Northeast, South, Northwest, Southwest of China all accounted for 14.51% of the patients, while patients with onset/awareness age >5 years made up 7.40% of the whole patient group, and patients with mild and moderate HL comprised 9.05% of the patients (Table 1). These figures implied that more subjects should be included from these subgroups in future investigations to enhance our knowledge of the HHL in these populations.

### De novo variants identified in AD and X-linked HL genes

In the present study, of the 36 diagnosed patients caused by variations in AD or X-linked HL genes, 20 patients were identified as being caused by 19 de novo variations in 7 genes. For example, five variants in *PTPN11* gene and three variants in *CHD7* gene detected in this study were all de novo mutations. Due to lack of probands and carrier status, prenatal screening for the de novo variants is not yet available clinically. But in 2019, a study reported the non-invasive prenatal screening for a panel of causative genes for frequent dominant monogenic diseases using circulating cell-free fetal DNA [41]. This approach provided sensitivity, specificity at levels sufficient to be transferred to the clinical practice for screening of this type of variant.

## Conclusion

In this study, 57.25% of the patient group had obtained positive molecular diagnoses, with 35 causative genes being involved. Of the 224 variants identified in the diagnosed patients, 83.04% (186/224) were related to AR inheritance, 12.95% (29/224) were related to AD, 3.12% (7/224) were related to X-linked, and 0.89% (2/224) were related to mitochondrial inheritance. Still, another 4.67% (48/1027) of the patients were categorized as uncertain diagnoses with at least one VUS, which indicated that more strategies were required to classify the VUS.

## Supplementary information

Supplementary Figures and Tables

## Data Availability

The data are available from the corresponding author on reasonable request.

## References

[1] Wilson BS, Tucci DL, Merson MH, O’Donoghue GM (2017). Global hearing health care: new findings and perspectives. Lancet..

[2] Li CM, Zhang X, Hoffman HJ, Cotch MF, Themann CL, Wilson MR (2014). Hearing impairment associated with depression in US adults, National Health and Nutrition Examination Survey 2005-2010. JAMA Otolaryngol Head Neck Surg.

[3] Contrera KJ, Betz J, Genther DJ, Lin FR (2015). Association of hearing impairment and mortality in the national health and nutrition examination survey. JAMA Otolaryngol Head Neck Surg.

[4] Stika CJ, Eisenberg LS, Johnson KC, Henning SC, Colson BG, Ganguly DH (2015). Developmental outcomes of early-identified children who are hard of hearing at 12 to 18 months of age. Early Hum Dev..

[5] Cunningham LL, Tucci DL (2017). Hearing loss in adults. N. Engl J Med.

[6] Sheffield AM, Smith RJH (2019). The Epidemiology of Deafness. Cold Spring Harb Perspect.

[7] Morton CC, Nance WE (2006). Newborn hearing screening-a silent revolution. N. Engl J Med.

[8] Bademci G, Foster J, Mahdieh N, Bonyadi M, Duman D, Cengiz FB (2016). Comprehensive analysis via exome sequencing uncovers genetic etiology in autosomal recessive non-syndromic deafness in a large multiethnic cohort. Genet Med..

[9] Yan D, Tekin D, Bademci G, Foster J, Cengiz FB, Kannan-Sundhari A (2016). Spectrum of DNA variants for non-syndromic deafness in a large cohort from multiple continents. Hum Genet.

[10] Li M, Nishio SY, Naruse C, Riddell M, Sapski S, Katsuno T (2020). Digenic inheritance of mutations in EPHA2 and SLC26A4 in Pendred syndrome. Nat Commun.

[11] Schrauwen I, Chakchouk I, Acharya A, Liaqat K, Irfanullah (2018). Novel digenic inheritance of PCDH15 and USH1G underlies profound non-syndromic hearing impairment. BMC Med Genet.

[12] Leone MP, Palumbo P, Ortore R, Castellana S, Palumbo O, Melchionda S (2017). Putative TMPRSS3/GJB2 digenic inheritance of hearing loss detected by targeted resequencing. Mol Cell Probes.

[13] Ołdak M, Lechowicz U, Pollak A, Oziębło D, Skarżyński H (2019). Overinterpretation of high throughput sequencing data in medical genetics: first evidence against TMPRSS3/GJB2 digenic inheritance of hearing loss. J Transl Med.

[14] Le Quesne Stabej P, Saihan Z, Rangesh N, Steele-Stallard HB, Ambrose J, Coffey A (2012). Comprehensive sequence analysis of nine Usher syndrome genes in the UK National Collaborative Usher Study. J Med Genet.

[15] Shearer AE, DeLuca AP, Hildebrand MS, Taylor KR, Gurrola J, Scherer S (2010). Comprehensive genetic testing for hereditary hearing loss using massively parallel sequencing. Proc Natl Acad Sci USA.

[16] Sloan-Heggen CM, Smith RJ (2016). Navigating genetic diagnostics in patients with hearing loss. Curr Opin Pediatr.

[17] Shearer AE, Smith RJ (2015). Massively parallel sequencing for genetic diagnosis of hearing loss: the new standard of care. Otolaryngol Head Neck Surg.

[18] Fang Y, Gu M, Wang C, Suo F, Wang G, Xia Y (2015). GJB2 as well as SLC26A4 gene mutations are prominent causes for congenital deafness. Cell Biochem Biophys.

[19] Xiang YB, Tang SH, Li HZ, Xu CY, Ch C, Xu YZ (2019). Mutation analysis of common deafness-causing genes among 506 patients with non-syndromic hearing loss from Wenzhou city, China. Int J Pediatr Otorhinolaryngol.

[20] Yang T, Guo L, Wang L, Yu X (2019). Diagnosis, intervention, and prevention of genetic hearing loss. Adv Exp Med Biol.

[21] Alford RL, Arnos KS, Fox M, Lin JW, Palmer CG, Pandya A (2014). American College of Medical Genetics and Genomics guideline for the clinical evaluation and etiologic diagnosis of hearing loss. Genet Med.

[22] Vila PM, Lieu JE (2015). Asymmetric and unilateral hearing loss in children. Cell Tissue Res.

[23] Lin PH, Hu CJ, Lin YH, Lee HY, Wu CC (2017). Etiologic and audiologic characteristics of patients with pediatric-onset unilateral and asymmetric sensorineural hearing loss. JAMA Otolaryngol Head Neck Surg.

[24] Barona R, Vizcaino JA, Krstulovic C, Barona L, Comeche C, Montalt J (2019). Does asymmetric hearing loss affect the ability to understand in noisy environments?. J Int Adv Otol.

[25] Richards S, Aziz N, Bale S, Bick D, Das S, Gastier-Foster J (2015). Standards and guidelines for the interpretation of sequence variants: a joint consensus recommendation of the American College of Medical Genetics and Genomics and the Association for Molecular Pathology. Genet Med.

[26] Oza AM, DiStefano MT, Hemphill SE, Cushman BJ, Grant AR, Siegert RK (2018). Expert specification of the ACMG/AMP variant interpretation guidelines for genetic hearing loss. Hum Mutat.

[27] Danial-Farran N, Brownstein Z, Gulsuner S, Tammer L, Khayat M, Aleme O (2018). Genetics of hearing loss in the Arab population of Northern Israel. Eur J Hum Genet.

[28] Gaildrat P, Killian A, Martins A, Tournier I, Frébourg T, Tosi M (2010). Use of Splicing Reporter Minigene Assay to Evaluate the Effect on Splicing of Unclassified Genetic Variants. Methods Mol Biol.

[29] Chen S, Dong C, Wang Q, Zhong Z, Qi Y, Ke X (2016). Targeted next-generation sequencing successfully detects causative genes in Chinese patients with hereditary hearing loss. Genet Test Mol Biomark.

[30] Sloan-Heggen CM, Bierer AO, Shearer AE, Kolbe DL, Nishimura CJ, Frees KL (2016). Comprehensive genetic testing in the clinical evaluation of 1119 patients with hearing loss. Hum Genet.

[31] Sennaroglu L, Bajin MD (2017). Classification and current management of inner ear malformations. Balk Med J.

[32] Ocak E, Duman D, Tekin M (2019). Genetic causes of inner ear anomalies: a review from the Turkish study group for inner ear anomalies. Balk Med J..

[33] Kari E, Llaci L, Go JL, Naymik M, Knowles JA, Leal SM (2020). Genes implicated in rare congenital inner ear and cochleovestibular nerve malformations. Ear Hear.

[34] Seligman KL, Shearer AE, Frees K, Nishimura C, Kolbe D, Dunn C (2022). Genetic Causes of Hearing Loss in a Large Cohort of Cochlear Implant Recipients. Otolaryngol Head Neck Surg.

[35] Abu Rayyan A, Kamal L, Casadei S, Brownstein Z, Zahdeh F, Shahin H (2020). Genomic analysis of inherited hearing loss in the Palestinian population. Proc Natl Acad Sci USA.

[36] Budde BS, Aly MA, Mohamed MR, Breß A, Altmüller J, Motameny S (2020). Comprehensive molecular analysis of 61 Egyptian families with hereditary nonsyndromic hearing loss. Clin Genet.

[37] Han JJ, Nguyen PD, Oh DY, Han JH, Kim AR, Kim MY (2019). Elucidation of the unique mutation spectrum of severe hearing loss in a Vietnamese pediatric population. Sci Rep.

[38] Safka Brozkova D, Poisson Marková S, Mészárosová AU, Jenčík J, Čejnová V, Čada Z (2020). Spectrum and frequencies of non GJB2 gene mutations in Czech patients with early non-syndromic hearing loss detected by gene panel NGS and whole-exome sequencing. Clin Genet.

[39] Bowl MR, Brown SDM (2018). Genetic landscape of auditory dysfunction. Hum Mol Genet.

[40] Kremer H (2019). Hereditary hearing loss; about the known and the unknown. Hear Res.

[41] Zhang J, Li J, Saucier JB, Feng Y, Jiang Y, Sinson J (2019). Non-invasive prenatal sequencing for multiple Mendelian monogenic disorders using circulating cell-free fetal DNA. Nat Med.

