# Supplementary material for: Molecular diagnose of a large hearing loss population from China by targeted genome sequencing
Source: J Hum Genet. 2022 Aug 19;67(11):643–9. doi: 10.1038/s10038-022-01066-5 (PMC9592555; doi:10.1038/s10038-022-01066-5)
Supplement: Supplementary file 1 — Supplementary Figures and Tables [file 10038_2022_1066_MOESM1_ESM.docx]

Supplementary Figures and Tables

**“Molecular diagnose of a large hearing loss population from China by targeted genome sequencing”**

Jie Wu^1,†^, Zongfu Cao^2,†^, Yu Su^1,†^, Yang Wang^3^, Ruikun Cai^2^, Jiyue Chen^1^, Bo Gao^1^, Mingyu Han^1^, Xiaohong Li^4^, DeJun Zhang^5^, Xue Gao^6^, Shasha Huang^1^, Quanfei Huang^3^, Yongyi Yuan^1,*^,Xu Ma^2,*^，Pu Dai^1,*^

^1^ Key Lab of Hearing Impairment Science of Ministry of Education, Key Lab of Hearing Impairment Prevention and Treatment of Beijing, National Clinical Research Center for Otolaryngologic Diseases, College of Otolaryngology Head and Neck Surgery, Chinese PLA General Hospital, Chinese PLA Medical School, #28 Fuxing Road, Beijing, 100853, China.

^2^ National Research Institute for Family Planning, National Human Genetic Resource Center, No. #12 Dahuisi Road, Beijing 100081, China.

^3^ Capital Bio Genomics Co. Dongguan, No. #1 Taoyuan Road, Dongguan City, Guangdong, 523808, China.

^4^ Department of Otolaryngology Head and Neck Surgery, Beijing Children’s Hospital, National Center for children’s Health, #56 South Lishi Road, Xicheng District, Beijing, 100045, China.

^5^ Department of Otolaryngology Head and Neck Surgery, The Second Hospital of Jilin University, #218 Ziqiang Street, Nanguan District, Changchun City, Jilin, 130041, China.

^6^ Department of Otolaryngology, PLA Rocket Force Characteristic Medical Center, #16 XinWai Da Jie, Beijing, 100088, China

^*^**Correspondence:**[daipu301@vip.sina.com(P.D.)](mailto:daipu301@vip.sina.com(P.D.));[yyymzh@163.com(Y.Y.)](mailto:yyymzh@163.com(Y.Y.));[maxubioinfo@163.com](mailto:maxubioinfo@163.com)(X.M.)

^†^ These authors contributed equally to this article.

**Content**

Table S1. The 227 HL-related genes included in the testing panel.

Table S2. Causative variants identified in *GJB2* (NM_004004.6) in this study.

Table S3. Causative variants identified in *SLC26A4* (NM_000441.2) in this study.

Table S4. Clinical information of patients caused by variations in *GJB2* and *SLC26A4*

Table S5. Diagnosed patients related to uncommon HL genes in this study.

Table S6. Uncertainly diagnosed patients identified in this study.

Table S7. The clinical phenotype information of diagnosed and undiagnosed patients.

Table S8. The information of 28 undiagnosed patients with inner malformation based on CT imaging. Figure S1. The inner ear malformations identified in this study.

Table S1. The 227 HL-related genes included in the testing panel

| Gene | Loci | Transcript | Exons | MIM |
| --- | --- | --- | --- | --- |
| Autosomal recessive non-syndromic HL/ARNSHL genes (60) | | | | |
| *GJB2* | DFNB1A/DFNA3A | NM_004004.6 | 2 | 121011 |
| *GJB6* | DFNB1B/DFNA3B | NM_006783.4 | 3 | 604418 |
| *MYO7A* | DFNB2/DFNA11/USH1B | NM_000260.4 | 49 | 275903 |
| *MYO15A* | DFNB3 | NM_016239.4 | 65 | 602666 |
| *SLC26A4* | DFNB4 | NM_000441.2 | 21 | 605646 |
| *TMIE* | DFNB6 | NM_147196.2 | 4 | 607237 |
| *TMC1* | DFNB7/11/DFNA36 | NM_138691.2 | 24 | 606706 |
| *TMPRSS3* | DFNB8/10 | NM_024022.3 | 13 | 605511 |
| *OTOF* | DFNB9 | NM_194248.3 | 47 | 603681 |
| *CDH23* | DFNB12/USH1D | NM_022124.6 | 68 | 605516 |
| *GIPC3* | DFNB15/72/95 | NM_133261.3 | 6 | 608792 |
| *STRC* | DFNB16 | NM_153700.2 | 29 | 606440 |
| *USH1C* | DFNB18/USH1C | NM_153676.4 | 27 | 605242 |
| *OTOG* | DFNB18B | NM_001277269.1 | 55 | 604487 |
| *TECTA* | DFNB21/DFNA8/12 | NM_005422.2 | 23 | 602574 |
| *OTOA* | DFNB22 | NM_144672.4 | 29 | 607038 |
| *PCDH15* | DFNB23/USH1F | NM_033056.4 | 33 | 6055514 |
| *RDX* | DFNB24 | NM_002906.3 | 14 | 179410 |
| *GRXCR1* | DFNB25 | NM_001080476.2 | 4 | 613283 |
| *TRIOBP* | DFNB28 | NM_001039141.3 | 24 | 609761 |
| *CLDN14* | DFNB29 | NM_144492.3 | 3 | 605608 |
| *MYO3A* | DFNB30 | NM_017433.5 | 35 | 606808 |
| *WHRN* | DFNB31/USH2D | NM_015404.4 | 12 | 607928 |
| *CDC14A* | DFNB32/105 | NM_033312.2 | 15 | 603504 |
| *ESRRB* | DFNB35 | NM_004452.3 | 11 | 602167 |
| *ESPN* | DFNB36 | NM_031475.3 | 13 | 606351 |
| *MYO6* | DFNB37/DFNA22 | NM_004999.4 | 35 | 600970 |
| *HGF* | DFNB39 | NM_000601.6 | 18 | 142409 |
| *ILDR1* | DFNB42 | NM_001199799.2 | 8 | 609739 |
| *ADCY1* | DFNB44 | NM_021116.4 | 20 | 103072 |
| *CIB2* | DFNB48 | NM_006383.4 | 6 | 605564 |
| *MARVELD2* | DFNB49 | NM_001038603.3 | 7 | 610572 |
| *COL11A2* | DFNB53/DFNA13/STL3 | NM_080680.2 | 66 | 120290 |
| *PDZD7* | DFNB57 | NM_001195263.2 | 17 | 612971 |
| *PJVK* | DFNB59 | NM_001042702.4 | 7 | 610219 |
| *SLC26A5* | DFNB61 | NM_198999.3 | 20 | 604943 |
| *LRTOMT* | DFNB63 | NM_001145308.4 | 7 | 612414 |
| *DCDC2* | DFNB66 | NM_016356.5 | 10 | 605755 |
| *LHFPL5* | DFNB66/67 | NM_182548.4 | 4 | 609427 |
| *S1PR2* | DFNB68 | NM_004230.4 | 2 | 605111 |
| *PNPT1* | DFNB70 | NM_033109.5 | 28 | 610316 |
| *BSND* | DFNB73 | NM_057176.3 | 4 | 606412 |
| *MSRB3* | DFNB74 | NM_001031679.3 | 7 | 613719 |
| *SYNE4* | DFNB76 | NM_001039876.3 | 8 | 615535 |
| *LOXHD1* | DFNB77 | NM_144612.6 | 40 | 613072 |
| *TPRN* | DFNB79 | NM_001128228.3 | 4 | 613354 |
| *GPSM2* | DFNB82 | NM_013296.5 | 15 | 609245 |
| *PTPRQ* | DFNB84/DFNA73 | NM_001145026.2 | 42 | 603317 |
| *OTOGL* | DFNB84 | NM_173591.3 | 58 | 614925 |
| *TBC1D24* | DFNB86/DFNA65 | NM_001199107.2 | 8 | 613577 |
| *ELMOD3* | DFNB88 | NM_001135022.2 | 14 | 615427 |
| *KARS* | DFNB89 | NM_001130089.1 | 15 | 601421 |
| *SERPINB6* | DFNB91 | NM_004568.5 | 7 | 173321 |
| *CABP2* | DFNB93 | NM_016366.3 | 7 | 607314 |
| *MET* | DFNB97 | NM_000245.4 | 21 | 164860 |
| *TSPEAR* | DFNB98 | NM_144991.3 | 12 | 612920 |
| *GRXCR2* | DFNB101 | NM_001080516.1 | 3 | 615752 |
| *EPS8* | DFNB102 | NM_004447.6 | 21 | 600206 |
| *CLIC5* | DFNB103 | NM_001114086.2 | 6 | 607293 |
| *FAM65B* | DFNB104 | NM_014722.5 | 23 | 611410 |
| Autosomal dominant non-syndromic HL/ ADNSHL genes (27) | | | | |
| *DIAPH1* | DFNA1 | NM_005219.5 | 28 | 602121 |
| *KCNQ4* | DFNA2A | NM_004700.4 | 14 | 603537 |
| *GJB3* | DFNA2B | NM_024009.3 | 2 | 603324 |
| *IFNLR1* | DFNA2C | NM_173064.3 | 7 | 607404 |
| *MYH14* | DFNA4A | NM_024729.3 | 41 | 608568 |
| *CEACAM16* | DFNA4B | NM_001039213.4 | 7 | 614591 |
| *GSDME* | DFNA5 | NM_004403.3 | 10 | 608798 |
| *WFS1* | DFNA6/14/38 | NM_006005.3 | 8 | 606201 |
| *COCH* | DFNA9 | NM_004086.3 | 12 | 603196 |
| *EYA4* | DFNA10 | NM_004100.5 | 20 | 603550 |
| *POU4F3* | DFNA15 | NM_002700.3 | 2 | 602460 |
| *MYH9* | DFNA17 | NM_002473.5 | 41 | 160775 |
| *ACTG1* | DFNA20/26 | NM_001614.5 | 6 | 102560 |
| *SIX1* | DFNA23/BOR3 | NM_005982.4 | 2 | 601205 |
| *SLC17A8* | DFNA25 | NM_139319.3 | 12 | 607557 |
| *GRHL2* | DFNA28 | NM_024915.4 | 16 | 608576 |
| *NLRP3* | DFNA34 | NM_004895.4 | 9 | 606416 |
| *COL11A1* | DFNA37/STL2 | NM_001854.4 | 67 | 120280 |
| *CRYM* | DFNA40 | NM_001888.5 | 10 | 123740 |
| *P2RX2* | DFNA41 | NM_174873.3 | 12 | 600844 |
| *CCDC50* | DFNA44 | NM_178335.3 | 12 | 611051 |
| *TJP2* | DFNA51 | NM_004817.4 | 23 | 607709 |
| *TNC* | DFNA56 | NM_002160.4 | 28 | 187380 |
| *DIABLO* | DFNA64 | NM_019887.6 | 7 | 605219 |
| *OSBPL2* | DFNA67 | NM_144498.3 | 14 | 606731 |
| *HOMER2* | DFNA68 | NM_004839.4 | 9 | 604799 |
| *KITLG* | DFNA69 | NM_000899.5 | 10 | 184745 |
| X-link HL genes (5) | | | | |
| *PRPS1* | DFNX1 | NM_002764.3 | 7 | 311850 |
| *POU3F4* | DFNX2 | NM_000307.5 | 1 | 300039 |
| *SMPX* | DFNX4 | NM_014332.3 | 5 | 300226 |
| *AIFM1* | DFNX5 | NM_004208.4 | 16 | 300169 |
| *COL4A6* | DFNX6 | NM_001847.4 | 45 | 303631 |
| Syndromic HL genes (34) | | | | |
| *PAX3* | WS1 | NM_181457.4 | 8 | 606597 |
| *MITF* | WS2A | NM_000248.3 | 9 | 156845 |
| *SNAI2* | WS2D | NM_003068.5 | 3 | 602150 |
| *SOX10* | WS2E/WS4C | NM_006941.3 | 4 | 602229 |
| *EDNRB* | WS4A | NM_000115.5 | 8 | 131244 |
| *EDN3* | WS4B | NM_207034.3 | 5 | 131242 |
| *USH1G* | USH1G | NM_173477.5 | 3 | 602150 |
| *USH2A* | USH2A | NM_206933.3 | 72 | 608400 |
| *ADGRV1* | USH2C | NM_032119.4 | 90 | 602851 |
| *CLRN1* | USH3A | NM_174878.3 | 3 | 606397 |
| *HARS* | USH3B | NM_002109.6 | 13 | 142810 |
| *TCOF1* | TCOF1 | NM_000356.4 | 26 | 606847 |
| *POLR1D* | TCOF2 | NM_015972.4 | 2 | 613715 |
| *POLR1C* | TCOF3 | NM_203290.4 | 9 | 610060 |
| *COL2A1* | STL1 | NM_001844.5 | 54 | 120140 |
| *COL9A1* | STL4 | NM_001851.5 | 38 | 120210 |
| *COL9A2* | STL5 | NM_001852.4 | 32 | 120260 |
| *HSD17B4* | PRLTS1 | NM_000414.4 | 24 | 601860 |
| *HARS2* | PRLTS2 | NM_012208.4 | 13 | 600783 |
| *CLPP* | PRLTS3/DFNB81 | NM_006012.4 | 6 | 601119 |
| *LARS2* | PRLTS4 | NM_015340.4 | 22 | 604544 |
| *TWNK* | PRLTS5 | NM_021830.5 | 5 | 606075 |
| *FOXI1* |  | NM_012188.5 | 2 | 601093 |
| *KCNJ10* |  | NM_002241.5 | 2 | 602208 |
| *NDP* | NDP1 | NM_000266.4 | 3 | 300658 |
| *KCNQ1* | JLNS1 | NM_000218.2 | 16 | 607542 |
| *KCNE1* | JLNS2 | NM_000219.6 | 4 | 176261 |
| *SEMA3E* |  | NM_012431.3 | 17 | 608188 |
| *CHD7* |  | NM_017780.4 | 38 | 608892 |
| *EYA1* | BOR1 | NM_000503.6 | 18 | 601653 |
| *SIX5* | BOR2 | NM_175875.5 | 3 | 600963 |
| *COL4A3* |  | NM_000091.4 | 52 | 120070 |
| *COL4A4* |  | NM_000092.4 | 48 | 120131 |
| *COL4A5* |  | NM_000495.5 | 51 | 303630 |
| Other HL related genes (101) | | | | |
| *ABCD1* |  | NM_000033.4 | 10 | 300371 |
| *ABHD12* |  | NM_001042472.3 | 13 | 613599 |
| *ALMS1* |  | NM_015120.4 | 23 | 606844 |
| *ALX3* |  | NM_006492.3 | 4 | 606014 |
| *ALX4* |  | NM_021926.4 | 4 | 605420 |
| *ANKH* |  | NM_054027.6 | 12 | 605145 |
| *ATP2B2* |  | NM_001001331.4 | 23 | 108733 |
| *ATP6V1B1* |  | NM_001692.4 | 14 | 192132 |
| *ATP6V1B2* |  | NM_001693.4 | 14 | 606939 |
| *BCAP31* |  | NM_001139441.1 | 8 | 300398 |
| *BCOR* |  | NM_017745.6 | 15 | 300485 |
| *BCS1L* |  | NM_004328.5 | 9 | 603647 |
| *C5ORF42* |  | NM_023073.3 | 52 | 614571 |
| *CACNA1D* |  | NM_000720.4 | 49 | 114206 |
| *CD151* |  | NM_004357.5 | 9 | 602243 |
| *CHM* |  | NM_000390.4 | 15 | 300390 |
| *CISD2* |  | NM_001008388.5 | 3 | 611507 |
| *CLCN7* |  | NM_001287.6 | 25 | 602727 |
| *CLCNKA* |  | NM_004070.4 | 20 | 602024 |
| *CLCNKB* |  | NM_000085.4 | 20 | 602023 |
| *COL1A1* |  | NM_000088.3 | 51 | 120150 |
| *COLEC11* |  | NM_024027.4 | 7 | 612502 |
| *COQ6* |  | NM_182476.3 | 12 | 614647 |
| *DCAF17* |  | NM_025000.4 | 14 | 612515 |
| *DHODH* |  | NM_001361.5 | 9 | 126064 |
| *DIAPH3* |  | NM_001042517.2 | 28 | 614567 |
| *DNAJC3* |  | NM_006260.5 | 12 | 601184 |
| *DNMT1* |  | NM_001130823.3 | 41 | 126375 |
| *DSPP* |  | NM_014208.3 | 5 | 125485 |
| *ECM1* |  | NM_004425.4 | 10 | 602201 |
| *ERCC3* |  | NM_000122.1 | 15 | 133510 |
| *FGF10* |  | NM_004465.2 | 3 | 602115 |
| *FGF3* |  | NM_005247.4 | 3 | 164950 |
| *FGFR1* |  | NM_023110.2 | 18 | 136350 |
| *FGFR2* |  | NM_000141.4 | 18 | 176943 |
| *FGFR3* |  | NM_000142.4 | 18 | 134934 |
| *FLNA* |  | NM_001456.3 | 47 | 300017 |
| *FOXC1* |  | NM_001453.3 | 1 | 601090 |
| *FRAS1* |  | NM_025074.7 | 74 | 607830 |
| *FREM2* |  | NM_207361.6 | 24 | 608945 |
| *GALE* |  | NM_000403.4 | 12 | 606953 |
| *GATA3* |  | NM_001002295.2 | 6 | 131320 |
| *GJA1* |  | NM_000165.5 | 2 | 121014 |
| *GRIP1* |  | NM_021150.4 | 24 | 604597 |
| *HMX1* |  | NM_018942.3 | 2 | 142992 |
| *HOXA2* |  | NM_006735.4 | 2 | 604685 |
| *IARS2* |  | NM_018060.4 | 23 | 612801 |
| *IGF1* |  | NM_000618.5 | 4 | 147440 |
| *LHX3* |  | NM_014564.5 | 6 | 600577 |
| *LRP2* |  | NM_004525.3 | 79 | 600073 |
| *LRP5* |  | NM_002335.4 | 23 | 603506 |
| *MAF* |  | NM_005360.5 | 2 | 177075 |
| *MASP1* |  | NM_139125.3 | 11 | 600521 |
| *MED12* |  | NM_005120.3 | 45 | 300188 |
| *MGP* |  | NM_000900.5 | 4 | 154870 |
| *MYO1A* |  | NM_005379.4 | 28 | 601478 |
| *NF1* |  | NM_001042492.2 | 58 | 613113 |
| *NF2* |  | NM_000268.3 | 16 | 607379 |
| *NOTCH2* |  | NM_024408.4 | 34 | 600275 |
| *OSTM1* |  | NM_014028.4 | 6 | 607649 |
| *PDSS1* |  | NM_014317.5 | 12 | 607429 |
| *PEX1* |  | NM_000466.3 | 24 | 602136 |
| *PEX7* |  | NM_000288.4 | 10 | 601757 |
| *PHYH* |  | NM_006214.4 | 9 | 602026 |
| *PITX2* |  | NM_153427.2 | 5 | 601542 |
| *PLEKHM1* |  | NM_014798.3 | 12 | 611466 |
| *PLOD1* |  | NM_000302.4 | 19 | 153454 |
| *PLOD3* |  | NM_001084.5 | 19 | 603066 |
| *PMP22* |  | NM_000304.4 | 5 | 601097 |
| *POLD1* |  | NM_002691.4 | 27 | 174761 |
| *PRRX1* |  | NM_022716.4 | 4 | 167420 |
| *PTPN11* |  | NM_002834.4 | 16 | 176876 |
| *RECQL4* |  | NM_004260.3 | 22 | 603780 |
| *RMND1* |  | NM_017909.4 | 12 | 614917 |
| *SALL1* |  | NM_002968.2 | 3 | 602218 |
| *SALL4* |  | NM_020436.5 | 4 | 607343 |
| *SERAC1* |  | NM_032861.4 | 17 | 614725 |
| *SF3B4* |  | NM_005850.5 | 6 | 605593 |
| *SLC33A1* |  | NM_004733.4 | 6 | 603690 |
| *SLC4A11* |  | NM_032034.3 | 19 | 610206 |
| *SLITRK6* |  | NM_032229.3 | 2 | 609681 |
| *SNX10* |  | NM_001199835.1 | 7 | 614780 |
| *SOST* |  | NM_025237.3 | 2 | 605740 |
| *SOX9* |  | NM_000346.4 | 3 | 608160 |
| *SQSTM1* |  | NM_003900.5 | 8 | 601530 |
| *TBX1* |  | NM_080647.1 | 9 | 602054 |
| *TBX22* |  | NM_001109878.2 | 9 | 300307 |
| *TCIRG1* |  | NM_006019.4 | 20 | 604592 |
| *TCTN3* |  | NM_015631.6 | 14 | 613847 |
| *TFAP2A* |  | NM_001032280.3 | 7 | 107580 |
| *TGFB1* |  | NM_000660.7 | 7 | 190180 |
| *TIMM8A* |  | NM_004085.4 | 2 | 300356 |
| *TNFRSF11A* |  | NM_003839.4 | 10 | 603499 |
| *TNFRSF11B* |  | NM_002546.4 | 5 | 602643 |
| *TNFSF11* |  | NM_003701.4 | 5 | 602642 |
| *TP63* |  | NM_003722.5 | 14 | 603273 |
| *TRMU* |  | NM_018006.5 | 11 | 610230 |
| *TSHZ1* |  | NM_005786.6 | 2 | 614427 |
| *TWIST1* |  | NM_000474.4 | 2 | 601622 |
| *TYR* |  | NM_000372.5 | 5 | 606933 |
| *ZNF687* |  | NM_020832.3 | 9 | 610568 |

Genes that cause both AR non-syndromic HL, AD non-syndromic HL and syndromic HL are listed under ARNSHL including: *GJB2, GJB6, MYO7A, TMC1, CDH23, USH1C, TECTA, PCDH15, MYO3A, WHRN, MYO6, COL11A2, PTPRQ, TBC1D24*;

Genes that cause both ADNHSL and syndromic HL are listed under ADNSHL including: *SIX1, COL11A1*.

Table S2. Causative variants identified in *GJB2* (NM_004004.6) in this study

| Nucleotide | Protein | Functional  consequence | Allele frequency  of patient group | Allele frequency of control group | Inheritance pattern | Reference | ACMG classification |
| --- | --- | --- | --- | --- | --- | --- | --- |
| c.235del | p.Leu79Cysfs*3 | frameshift | 278/2054 | 10/1040 | AR | rs80338943 | P |
| c.299_300del | p.His100Argfs*14 | frameshift | 80/2054 | 6/1040 | AR | rs111033204 | P |
| c.176_191del | p.Gly59Alafs*18 | frameshift | 18/2054 | 0 | AR | rs750188782 | P |
| c.508_511dup | p.Ala171Glufs*40 | frameshift | 17/2054 | 0 | AR | rs773528125 | P |
| c.427C>T | p.Arg143Trp | missense | 11/2054 | 1/1040 | AR | rs80338948 | P |
| c.257C>G | p.Thr86Arg | missense | 8/2054 | 2/1040 | AR | rs1291519904 | P |
| c.35dup | p.Val13Cysfs*35 | frameshift | 7/2054 | 0 | AR | rs80338939 | P |
| c.139G>T | p.Glu47* | nonsense | 6/2054 | 0 | AR | rs104894398 | P |
| c.560_605dup | p.Cys202* | stop-gain | 5/2054 | 0 | AR | rs1566528185 | P |
| c.9G>A | p.Trp3* | nonsense | 4/2054 | 0 | AR | rs111033401 | P |
| c.598G>A | p.Gly200Arg | missense | 2/2054 | 0 | AR | rs786204597 | LP |
| c.35del | p.Gly12Valfs*2 | frameshift | 4/2054 | 0 | AR | rs786204597 | P |
| c.428G>A | p.Arg143Gln | missense | 1/2054 | 0 | AR | rs104894401 | LP |
| c.157T>A | p.Cys53Ser | missense | 1/2054 | 0 | AR | Novel | LP |
| c.313_326del | p.Lys105Glyfs*5 | frameshift | 1/2054 | 0 | AR | rs111033253 | P |
| c.-23+1G>A |  | splice | 1/2054 | 0 | AR | rs80338940 | P |
| c.399G>A | p.Trp133* | nonsense | 1/2054 | 0 | AR | rs777225786 | P |
| c.232G>A | p.Ala78Thr | missense | 1/2054 | 0 | AR | Novel | LP |
| c.109G>A | p.Val37Ile | missense | 98/2054 | 27/1040 | AR | rs72474224 | P |
| c.224G>A | p.Arg75Gln | missense | 1/2054 | 0 | AD | rs28931593 | P |

P: Pathogenic; LP: Likely pathogenic.

Table S3. Causative variants identified in *SLC26A4* (NM_000441.2) in this study

| Nucleotide | Protein | Functional  consequence | Allele frequency of patient group | Allele frequency of control group | Reference | ACMG classification |
| --- | --- | --- | --- | --- | --- | --- |
| c.919-2A>G |  | splice | 302/2054 | 12/1040 | rs111033313 | P |
| c.2168A>G | p.His723Arg | missense | 61/2054 | 4/1040 | rs121908362 | P |
| c.2027T>A | p.Leu676Gln | missense | 16/2054 | 1/1040 | rs111033318 | P |
| c.1174A>T | p.Asn392Tyr | missense | 18/2054 | 0 | rs201562855 | P |
| c.1975G>C | p.Val659Leu | missense | 13/2054 | 1/1040 | rs200455203 | P |
| c.1226G>A | p.Arg409His | missense | 12/2054 | 0 | rs111033305 | P |
| c.1229C>T | p.Thr410Met | missense | 12/2054 | 0 | rs111033220 | P |
| c.1707+5G>A |  | splice | 10/2054 | 0 | rs192366176 | P |
| c.589G>A | p.Gly197Arg | missense | 8/2054 | 0 | rs111033380 | P |
| c.916dupG | p.Val306Glyfs*24 | frameshift | 8/2054 | 0 | rs768245266 | P |
| c.1079C>T | p.Ala360Val | missense | 8/2054 | 0 | rs786204474 | P |
| c.235C>T | p.Arg79* | nonsense | 5/2054 | 0 | rs786204581 | P |
| c.281C>T | p.Thr94Ile | missense | 5/2054 | 0 | rs1057516953 | P |
| c.1520del | p.Leu507* | stop-gain | 5/2054 | 1/1040 | rs786204601 | P |
| c.1692dupA | p.Cys565MetfsTer9 | frameshift | 5/2054 | 0 | rs746427774 | P |
| c.1181_1183del | p.Phe394del | deletion | 4/2054 | 0 | rs777008062 | P |
| c.249G>A | p.Trp83* | nonsense | 3/2054 | 0 | rs1057516658 | P |
| c.439A>G | p.Met147Val | missense | 3/2054 | 0 | rs760413427 | LP |
| c.754T>C | p.Ser252Pro | missense | 3/2054 | 0 | rs1315422549 | LP |
| c.1238A>G | p.Gln413Arg | missense | 3/2054 | 0 | rs142498437 | P |
| c.1262A>C | p.Gln421Pro | missense | 3/2054 | 0 | rs201660407 | LP |
| c.1327G>C | p.Glu443Gln | missense | 3/2054 | 0 | Novel | LP |
| c.1343C>T | p.Ser448Leu | missense | 3/2054 | 0 | rs747076316 | P |
| c.1594A>C | p.Ser532Arg | missense | 3/2054 | 0 | Novel | LP |
| c.1673A>T | p.Asn558Ile | missense | 3/2054 | 0 | rs766206507 | P |
| c.1336C>T | p.Gln446* | nonsense | 3/2054 | 0 | rs397516416 | P |
| c.1547dupC | p.Ser517Phefs*10 | frameshift | 2/2054 | 0 | rs786204450 | P |
| c.2T>C | p.Met1Thr | missense | 2/2054 | 0 | rs111033302 | P |
| c.946G>T | p.Gly316* | nonsense | 2/2054 | 0 | rs1554357231 | P |
| c.1001+5G>C |  | splice | 2/2054 | 0 | rs780131226 | LP |
| c.1263+1G>A |  | splice | 2/2054 | 0 | rs1057517000 | P |
| c.1340delA | p.Lys447Serfs*8 | frameshift | 2/2054 | 0 | rs1562835515 | P |
| c.1595G>T | p.Ser532Ile | missense | 2/2054 | 0 | rs1057516243 | LP |
| c.1991C>T | p.Ala664Val | missense | 2/2054 | 0 | NA | LP |
| c.2089+1G>A |  | splice | 2/2054 | 0 | rs727503430 | P |
| c.3G>A | p.Met1Ile | missense | 1/2054 | 0 | Novel | P |
| c.79T>C | p.Tyr27His | missense | 1/2054 | 0 | Novel | LP |
| c.203T>C | p.Leu68Pro | missense | 1/2054 | 0 | rs749712560 | LP |
| c.269C>T | p.Ser90Leu | missense | 1/2054 | 0 | rs370588279 | LP |
| c.279T>A | p.Ser93Arg | missense | 1/2054 | 0 | Novel | LP |
| c.305-1G>A |  | splice | 1/2054 | 0 | Novel | P |
| c.334C>T | p.Pro112Ser | missense | 1/2054 | 0 | rs1409565648 | LP |
| c.387delC | p.Phe130Leufs*15 | frameshift | 1/2054 | 0 | NA | P |
| c.401G>C | p.Arg134Thr | missense | 1/2054 | 0 | Novel | LP |
| c.414delT | p.Gly139Aspfs*6 | frameshift | 1/2054 | 0 | rs1562822698 | P |
| c.415+2T>C |  | splice | 1/2054 | 0 | NA | P |
| c.415+7A>G |  | splice | 1/2054 | 0 | rs765884316 | LP |
| c.416G>T | p.Gly139Val | missense | 1/2054 | 0 | rs756272252 | LP |
| c.421T>C | p.Phe141Leu | missense | 1/2054 | 0 | novel | LP |
| c.563T>C | p.Ile188Thr | missense | 1/2054 | 0 | rs1205712508 | LP |
| c.578C>T | p.Thr193Ile | missense | 1/2054 | 0 | rs111033348 | LP |
| c.600+2T>C |  | splice | 1/2054 | 0 | Novel | P |
| c.624_632delinsACTTGGC | p.Gly209Leufs*50 | frameshift | 1/2054 | 0 | Novel | P |
| c.667_669dup | p.Phe223dup | duplication | 1/2054 | 0 | Novel | P |
| c.698_701del | p.Val233Aspfs*3 | frameshift | 1/2054 | 0 | Novel | P |
| c.716T>A | p.Val239Asp | missense | 1/2054 | 0 | rs111033256 | P |
| c.812A>G | p.Asp271Gly | missense | 1/2054 | 0 | Novel | LP |
| c.920C>T | p.Thr307Met | missense | 1/2054 | 0 | rs144691257 | LP |
| c.1001G>T | p.Gly334Val | missense&splice | 1/2054 | 0 | rs146281367 | LP |
| c.1002G>T | p.Gly334Gly | splice | 1/2054 | 0 | Novel | LP |
| c.1173C>A | p.Ser391Arg | missense | 1/2054 | 0 | rs1057517042 | LP |
| c.1318A>T | p.Lys440* | nonsense | 1/2054 | 0 | rs1562835480 | P |
| c.1325T>C | p.Leu442Pro | missense | 1/2054 | 0 | Novel | LP |
| c.1334T>G | p.Leu445Trp | missense | 1/2054 | 0 | rs111033307 | P |
| c.1342-1_1342insCTG |  | splice | 1/2054 | 0 | Novel | P |
| c.1343C>A | p.Ser448* | nonsense | 1/2054 | 0 | rs747076316 | P |
| c.1369A>G | p.Asn457Asp | missense | 1/2054 | 0 | Novel | LP |
| c.1522A>G | p.Thr508Ala | missense | 1/2054 | 0 | rs727505088 | LP |
| c.1552T>G | p.Trp518Gly | missense | 1/2054 | 0 | Novel | LP |
| c.1586T>G | p.Ile529Ser | missense | 1/2054 | 0 | rs786204739 | LP |
| c.1657C>T | p.Pro553Ser | missense | 1/2054 | 0 | Novel | LP |
| c.1716T>A | p.Phe572Leu | missense | 1/2054 | 0 | Novel | P |
| c.1746delG | p.Ala584Argfs*2 | frameshift | 1/2054 | 0 | rs1241745103 | P |
| c.1786C>T | p.Gln596* | nonsense | 1/2054 | 0 | rs1476190682 | P |
| c.1803+1G>C |  | splice | 1/2054 | 0 | Novel | P |
| c.1828delT | p.Ser610Glnfs*25 | frameshift | 1/2054 | 0 | Novel | P |
| c.1949T>A | p.Val650Asp | missense | 1/2054 | 0 | rs1057517161 | LP |
| c.1985G>A | p.Cys662Tyr | missense | 1/2054 | 0 | Novel | LP |
| c.1996T>C | p.Ser666Pro | missense | 1/2054 | 0 | rs397516425 | LP |
| c.2000T>C | p.Phe667Ser | missense | 1/2054 | 0 | Novel | LP |
| c.2039delT | p.Val680Alafs*9 | frameshift | 1/2054 | 0 | Novel | P |
| c.2086C>T | p.Gln696* | nonsense | 1/2054 | 0 | rs752807925 | P |
| c.2107C>G | p.Leu703Val | missense | 1/2054 | 0 | Novel | LP |
| c.2118C>A | p.Cys706* | nonsense | 1/2054 | 0 | rs142656144 | P |
| c.2167C>G | p.His723Asp | missense | 1/2054 | 0 | rs1417146153 | P |

NA: Not available.

Table S4. Clinical information of patients caused by variations in *GJB2* and *SLC26A4*

| Characteristic | Number | Percentage | Number | Percentage |
| --- | --- | --- | --- | --- |
|  | *GJB2* | | *SLC26A4* | |
| Age of onset/awareness(years) | | | | |
| 0 | 99 | 47.83% | 94 | 33.81% |
| >0 and ≤2 | 78 | 37.68% | 90 | 32.37% |
| >2 and ≤5 | 22 | 10.63% | 76 | 27.34% |
| >5 and ≤9 | 2 | 0.97% | 10 | 3.60% |
| >9 and ≤19 | 3 | 1.45% | 4 | 1.44% |
| >19 | 0 | 0.00% | 0 | 0.00% |
| NP | 3 | 1.45% | 4 | 1.44% |
| Severity | | | | |
| Mild | 2 | 0.97% | 2 | 0.72% |
| Moderate | 15 | 7.25% | 17 | 6.12% |
| Moderately Severe | 35 | 16.91% | 50 | 17.99% |
| Severe | 29 | 14.01% | 85 | 30.58% |
| Profound | 120 | 57.97% | 115 | 41.37% |
| NP | 6 | 2.90% | 9 | 3.24% |
| Symmetry | | | | |
| Symmetric | 165 | 79.71% | 80 | 28.78% |
| Asymmetric | 27 | 13.04% | 174 | 62.59% |
| NP | 15 | 7.25% | 24 | 8.63% |
| Family history | | | | |
| No | 182 | 87.92% | 249 | 89.57% |
| Yes | 25 | 12.08% | 29 | 10.43% |
| Pendred Syndrome |  |  |  |  |
| Mondini malformation |  |  | 235 | 84.53% |
| Goiter |  |  | 6 | 2.16% |
| NP |  |  | 39 | 14.03% |

NP: Not provided

Table S5. Diagnosed patients related to uncommon HL genes in this study

| Patient | Severity  of HL | Family history | Gene | Variant | Functional  consequence | Allele frequency of patient group | Allele frequency of control group | Zygosity | Inheritance  pattern | Reference | ACMG  classification |
| --- | --- | --- | --- | --- | --- | --- | --- | --- | --- | --- | --- |
| 8078 | NP | No | *POU3F4* | NM_000307.5:c.65_66del:p.Ser22Cysfs*19 | frameshift | 1/2054 | 0 | Hemi (*de novo*) | XLR | Novel | P |
| 8117 | Profound | No | *MYO15A* | NM_016239.4:c.6863C>T:p.Ser2288Leu | missense | 1/2054 | 0 | Het | AR | rs886052676 | LP |
|  |  |  | *MYO15A* | NM_016239.4:c.8828dup:p.Ser2945Phefs*55 | frameshift | 1/2054 | 0 | Het | AR | rs751628774 | P |
| 8119 | Profound | Yes | *MITF* | NM_198159.3:c.949del:p.Arg317Glufs*5 | frameshift | 1/2054 | 0 | Het (*de novo*) | AD | Novel | P |
| 8233 | Profound | No | *MITF* | NM_198159.3:c.1066C>T:p.Arg356* | nonsense | 1/2054 | 0 | Het | AD | rs1057517966 | P |
| 8286 | M S | No | *PJVK* | NM_001042702.5:c.532C>T:p.Arg178* | nonsense | 1/2054 | 0 | Het | AR | rs570669186 | P |
|  |  |  | *PJVK* | NM_001042702.5:c.547C>T:p.Arg183Trp | missense | 1/2054 | 0 | Het | AR | rs111706634 | LP |
| 8288 | M S | No | *TMC1* | NM_138691.3:c.236+1G>A | splice | 2/2054 | 0 | Het (*de novo*) | AR | rs775428246 | P |
|  |  |  | *TMC1* | NM_138691.3:c.589G>A:p.Gly197Arg | missense | 3/2054 | 0 | Het | AR | rs753687760 | LP |
| 8337 | NP | No | *POU3F4* | NM_000307.5:c.985C>G:p.Arg329Gly | missense | 1/2054 | 0 | Hemi (*de novo*) | XLR | Novel | LP |
| 8352 | Profound | Yes | *MYO15A* | NM_016239.4:c.7711_7712dup:p.Gln2571Hisfs*35 | frameshift | 1/2054 | 0 | Het | AR | rs1267868260 | P |
|  |  |  | *MYO15A* | NM_016239.4:c.10250_10252del:p.Ser3417del | deletion | 5/2054 | 1/1040 | Het | AR | rs760069953 | P |
| 8474 | Profound | No | *PCDH15* | NM_001142763.2:c.1021C>T:p.Arg341Ter | nonsense | 2/2054 | 0 | Hom | AR | rs370261904 | P |
| 8622 | NP | No | *MYO15A* | NM_016239.4:c.9400C>T:p.Arg3134* | nonsense | 1/2054 | 0 | Het | AR | rs1330631412 | P |
|  |  |  | *MYO15A* | NM_016239.4:c.10538_10544del:p.Leu3513Profs*39 | frameshift | 1/2054 | 0 | Het | AR | Novel | P |
| 8806 | Profound | No | *POU3F4* | NM_000307.5:c.669T>A:p.Tyr223* | nonsense | 1/2054 | 0 | Hemi (*de novo*) | XLR | Novel | P |
| 8926 | Profound | No | *MYO15A* | NM_016239.4:c.4596+1_4596+2del | splice | 1/2054 | 0 | Het | AR | Novel | P |
|  |  |  | *MYO15A* | NM_016239.4:c.10419_10423del:p.Ser3474Profs*42 | frameshift | 2/2054 | 0 | Het | AR | Novel | P |
| 8976 | Profound | No | *OTOF* | NM_194248.3:c.5570G>A:p.Gly1857Asp | missense | 1/2054 | 0 | Het | AR | Novel | LP |
|  |  |  | *OTOF* | NM_194248.3:c.5212_5214del:p.Ile1738del | deletion | 1/2054 | 0 | Het | AR | Novel | P |
| 9130 | Profound | No | *COL4A3* | NM_000091.5:c.2439del:p.Arg814Glyfs*9 | frameshift | 1/2054 | 0 | Het | AR | Novel | P |
|  |  |  | *COL4A3* | NM_000091.5:c.4318del:p.Thr1440Profs*89 | frameshift | 1/2054 | 0 | Het | AR | Novel | P |
| 9169 | Profound | No | *MYO15A* | NM_016239.4:c.6177+1G>T | splice | 4/2054 | 0 | Het | AR | rs751142446 | P |
|  |  |  | *MYO15A* | NM_016239.4:c.10258_10260del:p.Phe3420del | deletion | 1/2054 | 0 | Het | AR | rs1174503300 | LP |
| 9243 | Moderate | Yes | *WFS1* | NM_006005.3:c.2389G>A:p.Asp797Asn | missense | 1/2054 | 0 | Het | AD | rs1553879004 | LP |
| 9252 | Profound | Yes | *MITF* | NM_198159.3:c.937+1G>C | splice | 1/2054 | 0 | Het (*de novo*) | AD | Novel | P |
| 9352 | Profound | Yes | *MYO7A* | NM_000260.4:c.397C>G:p.His133Asp | missense | 1/2054 | 0 | Het | AR | rs111033403 | LP |
|  |  |  | *MYO7A* | NM_000260.4:c.1091dup:p.Asp365Argfs*8 | frameshift | 1/2054 | 0 | Het | AR | Novel | P |
| 9511 | Profound | No | *OTOF* | NM_194248.2:c.5000C>A:p.Ala1667Asp | missense | 3/2054 | 0 | Het | AR | rs1423274041 | LP |
|  |  |  | *OTOF* | NM_194248.2:c.1364_1366delinsTTGC:p.Tyr455Phefs*21 | frameshift | 1/2054 | 0 | Het | AR | Novel | P |
| 9576 | Profound | No | *MYO15A* | NM_016239.4:c.6177+1G>T | splice | 4/2054 | 0 | Het | AR | rs751142446 | P |
|  |  |  | *MYO15A* | NM_016239.4:c.6898A>T:p.Lys2300* | nonsense | 1/2054 | 0 | Het | AR | Novel | P |
| 9584 | Profound | No | *OTOF* | NM_194248.3:c.4718T>C:p.Ile1573Thr | missense | 1/2054 | 0 | Het | AR | rs111033405 5 | LP |
|  |  |  | *OTOF* | NM_194248.3:c.709C>T:p.Arg237* | nonsense | 4/2054 | 0 | Het | AR | rs397515610 | P |
| 9599-3 | M S | Yes | *TMC1* | NM_138691.3:c.150del:p.Asn50Lysfs*26 | frameshift | 1/2054 | 0 | Het | AR | Novel | P |
|  |  |  | *TMC1* | NM_138691.3:c.589G>A:p.Gly197Arg | missense | 3/2054 | 0 | Het | AR | rs753687760 | LP |
| 9601 | Profound | No | *PTPN11* | NM_002834.5:c.922A>G:p.Asn308Asp | missense | 1/2054 | 0 | Het (*de novo*) | AD | rs28933386 | LP |
| 9613 | Profound | No | *MITF* | NM_198159.3:c.952_954del:p.Arg318del | deletion | 2/2054 | 0 | Het (*de novo*) | AD | rs1553704814 | P |
| 9647 | Mild | Yes | *EYA4* | NM_001301013.2:c.804+2T>A | splice | 1/2054 | 0 | Het | AD | Novel | P |
| 9726 | Severe | Yes | *LARS2* | NM_015340.4:c.880G>A:p.Glu294Lys | missense | 2/2054 | 0 | Het | AR | rs749627411 | LP |
|  |  |  | *LARS2* | NM_015340.4:c.1783del:p.Ala595Profs*39 | frameshift | 1/2054 | 0 | Het | AR | Novel | P |
| 9831 | Profound | No | *OTOF* | NM_194248.3:c.5360G>A:p.Gly1787Asp | missense | 1/2054 | 0 | Het | AR | Novel | LP |
|  |  |  | *OTOF* | NM_194248.3:c.709C>T:p.Arg237* | nonsense | 4/2054 | 0 | Het | AR | rs397515610 | P |
| 9875 | Profound | No | *MYO15A* | NM_016239.4:c.212del:p.Lys71Serfs*17 | frameshift | 1/2054 | 0 | Het | AR | Novel | P |
|  |  |  | *MYO15A* | NM_016239.4:c.9941del:p.Tyr3314Serfs*9 | frameshift | 1/2054 | 0 | Het | AR | Novel | P |
| 9896 | Profound | No | *CDH23* | NM_022124.6:c.4562A>G:p.Asn1521Ser | missense | 1/2054 | 0 | Het | AR | rs780987516 | LP |
|  |  |  | *CDH23* | NM_022124.6:c.5924-1G>A | splice | 1/2054 | 0 | Het | AR | rs1841212177 | P |
| 10044 | Profound | No | *PCDH15* | NM_001142769.3:c.4766_4767del:p.Leu1589HisfsTer24 | frameshift | 1/2054 | 1/1040 | Het | AR | rs1554815781 | P |
|  |  |  | *PCDH15* | NM_001142763.2:c.3877T>C:p.Ser1293Pro | missense | 1/2054 | 0 | Het | AR | rs1554827269 | LP |
| 10050 | Severe | Yes | *MYH14* | NM_001145809.2:c.5990del:p.Thr1997ArgfsTer8 | frameshift | 1/2054 | 0 | Het | AD | Novel | P |
| 10052 | Profound | No | *MYO7A* | NM_000260.4:c.4398G>A:p.Trp1466* | nonsense | 1/2054 | 0 | Het | AR | rs1956759935 | P |
|  |  |  | *MYO7A* | NM_000260.4:c.6126C>G:p.Tyr2042* | nonsense | 1/2054 | 0 | Het | AR | Novel | P |
| 10123 | Profound | No | *MYO15A* | NM_016239.4:c.6956+9C>G | splice | 4/2054 | 0 | Het | AR | rs1482709090 | LP |
|  |  |  | *MYO15A* | NM_016239.4:c.10250_10252del:p.Ser3417del | deletion | 5/2054 | 1/1040 | Het | AR | rs760069953 | P |
| 10150 | Severe | Yes | *POU3F4* | NM_000307.5:c.877C>G:p.Leu293Val | missense | 1/2054 | 0 | Hemi | XLR | rs780027419 | LP |
| 10157 | Profound | No | *PTPN11* | NM_002834.5:c.854T>C:p.Phe285Ser | missense & splice | 1/2054 | 0 | Het (*de novo*) | AD | rs121918463 | LP |
| 10160 | Profound | No | *OTOF* | NM_194248.3:c.5000C>A:p.Ala1667Asp | missense | 3/2054 | 0 | Het | AR | rs1423274041 | LP |
|  |  |  | *OTOF* | NM_194248.3:c.1912+5G>C | splice | 1/2054 | 0 | Het | AR | Novel | LP |
| 10206 | Profound | No | *OTOF* | NM_194248.3:c.1962dup:p.Pro655Alafs*8 | frameshift | 1/2054 | 0 | Het | AR | Novel | P |
|  |  |  | *OTOF* | NM_194248.3:c.5000C>A:p.Ala1667Asp | missense | 3/2054 | 0 | Het | AR | rs1423274041 | LP |
| 10217 | Severe | No | *SMPX* | NM_014332.3:c.132+1G>A | splice | 1/2054 | 0 | Hemi | XLD | Novel | LP |
| 10218 | Profound | No | *TRIOBP* | NM_001039141.3:c.1783C>T:p.Arg595* | nonsense | 1/2054 | 0 | Het | AR | rs371412957 | P |
|  |  |  | *TRIOBP* | NM_001039141.3:c.2176C>T:p.Arg726* | nonsense | 1/2054 | 0 | Het (*de novo*) | AR | rs375857763 | P |
| 10248 | Moderate | Yes | *EYA4* | NM_001301013.2:c.804+2del | splice | 1/2054 | 0 | Het | AD | Novel | P |
| 10300 | M S | No | *MYO3A* | NM_017433.5:c.824G>A:p.Arg275His | missense | 4/2054 | 0 | Het | AR | rs928696768 | LP |
|  |  |  | *MYO3A* | NM_017433.5:c.3737_3738del:p.Glu1246Glyfs*5 | frameshift | 1/2054 | 0 | Het | AR | Novel | P |
| 10302 | Profound | No | *TMPRSS3* | NM_001256317.3:c.271C>T:p.Arg91* | nonsense | 4/2054 | 0 | Het | AR | rs199903164 | P |
|  |  |  | *TMPRSS3* | NM_001256317.3:c.147dup:p.Pro50Serfs*35 | frameshift | 1/2054 | 0 | Het | AR | Novel | P |
| 10309 | M S | Yes | *MYO3A* | NM_017433.5:c.824G>A:p.Arg275His | missense | 4/2054 | 0 | Hom | AR | rs928696768 | LP |
| 10382 | Severe | No | *TRIOBP* | NM_001039141.3:c.2932_2956del:p.Ser978Argfs*19 | frameshift | 2/2054 | 0 | Het | AR | rs1478055831 | P |
|  |  |  | *TRIOBP* | NM_001039141.3:c.3672_3673del:p.Arg1225Serfs*11 | frameshift | 1/2054 | 0 | Het | AR | Novel | P |
| 10393 | M S | Yes | *USH2A* | NM_206933.4:c.8714dup:p.His2905Glnfs*33 | frameshift | 1/2054 | 0 | Het | AR | Novel | P |
|  |  |  | *USH2A* | NM_206933.4:c.99_100insT:p.Arg34SerfsTer41 | frameshift | 2/2054 | 1/1040 | Het | AR | rs141672841 | P |
| 10442 | Profound | Yes | *PAX3* | NM_001127366.3:c.664C>T:p.Arg222* | nonsense | 1/2054 | 0 | Het | AD | rs772241382 | P |
| 10482 | Profound | No | *MYO15A* | NM_016239.4:c.3524dup:p.Ser1176Valfs*14 | frameshift | 3/2054 | 0 | Hom | AR | rs766187994 | LP |
| 10526 | Profound | Yes | *MYO15A* | NM_016239.4:c.6045A>T:p.Ala2015ALa | splice | 1/2054 | 0 | Het | AR | Novel | LP |
|  |  |  | *MYO15A* | NM_016239.4:c.6328G>A:p.Gly2110Arg | missense | 1/2054 | 0 | Het | AR | rs756396137 | LP |
| 10541 | Profound | Yes | *PTPN11* | NM_002834.5:c.1510A>G:p.Met504Val | missense | 1/2054 | 0 | Het (*de novo*) | AD | rs397507547 | LP |
| 10586 | Severe | No | *PTPN11* | NM_002834.5:c.1471C>T:p.Pro491Ser | missense | 1/2054 | 0 | Het (*de novo*) | AD | rs397507539 | LP |
| 10611 | NP | No | *MYO15A* | NM_016239.4:c.6892C>T:p.Arg2298* | nonsense | 1/2054 | 0 | Het | AR | rs900747631 | P |
|  |  |  | *MYO15A* | NM_016239.4:c.7654+1G>A | splice | 1/2054 | 0 | Het | AR | Novel | P |
| 10615 | Profound | No | *MITF* | NM_198159.3:c.1180C>T:p.Arg394Ter | nonsense | 1/2054 | 0 | Het | AD | Novel | LP |
| 10642 | Severe | No | *POU3F4* | NM_000307.5:c.609_610del:p.Arg204LysfsTer21 | frameshift | 1/2054 | 0 | Hemi | XLR | Novel | P |
| 10657 | Profound | No | *TMIE* | NM_147196.3:c.144_145del:p.Val49Glyfs*65 | frameshift | 1/2054 | 0 | Het | AR | Novel | P |
|  |  |  | *TMIE* | NM_147196.3:c.458_462del:p.Lys153Argfs*116 | frameshift | 1/2054 | 0 | Het | AR | rs1349277131 | P |
| 10711 | Profound | No | *PAX3* | NM_001127366.3:c.781C>T:p.Arg261* | nonsense | 1/2054 | 0 | Het (*de novo*) | AD | rs886041319 | P |
| 10740 | Profound | Yes | *PAX3* | NM_001127366.3:c.143del:p.Gly48Alafs*62 | frameshift | 1/2054 | 0 | Het | AD | rs1695346408 | P |
| 10750 | Profound | No | *MITF* | NM_198159.3:c.952_954del:p.Arg318del | deletion | 2/2054 | 0 | Het (*de novo*) | AD | rs1553704814 | P |
| 10771 | Profound | No | *MYO15A* | NM_016239.4:c.4519C>T:p.Arg1507* | nonsense | 2/2054 | 0 | Het | AR | rs549138385 | P |
|  |  |  | *MYO15A* | NM_016239.4:c.6177+1G>T | splice | 4/2054 | 0 | Het | AR | rs751142446 | P |
| 10834 | Profound | No | *MYO15A* | NM_016239.4:c.5193_5194insACAG:p.Val1732Thrfs*78 | frameshift | 1/2054 | 0 | Het | AR | Novel | P |
|  |  |  | *MYO15A* | NM_016239.4:c.8340+5G>A | splice | 1/2054 | 0 | Het | AR | rs752773977 | LP |
| 10840 | Profound | No | *TIMM8A* | NM_004085.4:c.232_233insCAAT:p.Leu78SerfsTer21 | frameshift | 1/2054 | 0 | Hemi | XLR | Novel | LP |
| 10865 | M S | No | *USH2A* | NM_206933.4:c.7184_7194del:p.Leu2395Hisfs*19 | frameshift | 1/2054 | 0 | Het | AR | Novel | P |
|  |  |  | *USH2A* | NM_206933.4:c.538T>C:p.Ser180Pro | missense | 1/2054 | 0 | Het | AR | rs1171672823 | LP |
| 10921 | Moderate | Yes | *MYO6* | NM_004999.4:c.826C>T:p.Arg276Ter | nonsense | 1/2054 | 0 | Het | AD | rs727503326 | P |
| 10990 | M S | No | *MYH9* | NM_002473.6:c.3943-8G>T | splice | 1/2054 | 0 | Het (*de novo*) | AD | rs760886457 | LP |
| 11051 | M S | No | *EYA1* | NM_000503.6:c.880C>T:p.Arg294Ter | nonsense | 1/2054 | 0 | Het (*de novo*) | AD | rs1816578250 | P |
| 11207 | M S | Yes | *GSDME* | NM_004403.3:c.991-15_991-13delTTC | Splice | 1/2054 | 0 | Het | AD | rs727505273 | P |
| 11237 | M S | Yes | *GATA3* | NM_001002295.2:c.352_353del:p.Ser118GlnfsTer185 | frameshift | 1/2054 | 0 | Het | AD | Novel | LP |
| 11242 | Profound | No | *LARS2* | NM_015340.4:c.41_42del:p.Leu14ProfsTer46 | frameshift | 1/2054 | 0 | Het | AR | Novel | P |
|  |  |  | *LARS2* | NM_015340.4:c.880G>A:p.Glu294Lys | missense | 2/2054 | 0 | Het | AR | rs749627411 | P |
| 11387 | Moderate | No | *CLRN1* | NM_001195794.1:c.736T>C:p.Ter246ArgextTer22 | stop-loss | 1/2054 | 0 | Het | AR | Novel | LP |
|  |  |  | *CLRN1* | NM_001195794.1:c.658C>T:p.Arg220Ter | nonsense | 1/2054 | 0 | Het | AR | rs373208120 | P |
| 11431 | Profound | No | *OTOF* | NM_194248.3:c.5108_5114delinsTCTTCCTGGG:p.Arg1703_Glu1705delinsLeuPheLeuGly | delins | 1/2054 | 0 | Het | AR | Novel | P |
|  |  |  | *OTOF* | NM_194248.3:c.709C>T:p.Arg237* | nonsense | 4/2054 | 0 | Het | AR | rs397515610 | P |
| 11452 | M S | No | *OTOA* | NM_001161683.2:c.1115G>A:p.Gly372Asp | missense | 2/2054 | 0 | Hom | AR | rs200656442 | LP |
| 11456 | Profound | No | *MYO15A* | NM_016239.4:c.1661del:p.Gly554Alafs*75 | frameshift | 1/2054 | 0 | Het | AR | Novel | P |
|  |  |  | *MYO15A* | NM_016239.4:c.8183G>A:p.Arg2728His | missense | 1/2054 | 0 | Het | AR | rs184435771 | LP |
| 11813 | Profound | No | *LARS2* | NM_015340.4:c.556C>T:p.Gln186* | nonsense | 1/2054 | 0 | Het | AR | rs1163473923 | P |
|  |  |  | *LARS2* | NM_015340.4:c.1886C>T:p.Thr629Met | missense | 1/2054 | 0 | Het | AR | rs398123036 | LP |
| 11876 | M S | No | *USH2A* | NM_206933.4:c.14038C>T:p.Gln4680* | nonsense | 1/2054 | 0 | Het | AR | rs1657798642 | P |
|  |  |  | *USH2A* | NM_206933.4:c.11156G>A:p.Arg3719His | missense | 1/2054 | 0 | Het | AR | rs527236139 | LP |
| 11995 | Profound | No | *MITF* | NM_198159.3:c.910A>G:p.Arg304Gly | missense | 1/2054 | 0 | Het (*de novo*) | AD | Novel | LP |
| 12780 | Profound | No | *EYA1* | NM_000503.6:c.1597+1G>C | splice | 1/2054 | 0 | Het (*de novo*) | AD | rs1563630117 | P |
| 12837 | Profound | No | *CHD7* | NM_017780.4:c.6217C>T:p.Gln2073* | nonsense | 1/2054 | 0 | Het (*de novo*) | AD | Novel | P |
| 12848 | Profound | No | *MYO15A* | NM_016239.4:c.6956+9C>G | splice | 4/2054 | 0 | Het | AR | rs1482709090 | LP |
|  |  |  | *MYO15A* | NM_016239.4:c.9690+1G>A | splice | 2/2054 | 0 | Het | AR | Novel | P |
| 12852 | Profound | No | *LOXHD1* | NM_144612.6:c.5888del:p.Gly1963AlafsTer136 | frameshift | 2/2054 | 0 | Het | AR | rs1442485603 | P |
|  |  |  | *LOXHD1* | NM_144612.6:c.6355del:p.Ala2119ProfsTer10 | frameshift | 1/2054 | 0 | Het (*de novo*) | AR | Novel | P |
| 12929 | Profound | No | *TMC1* | NM_138691.3:c.100C>T:p.Arg34* | nonsense | 1/2054 | 0 | Het | AR | rs121908073 | P |
|  |  |  | *TMC1* | NM_138691.3:c.1810C>T:p.Arg604* | nonsense | 1/2054 | 0 | Het | AR | rs777777359 | P |
| 12940 | Severe | No | *CHD7* | NM_017780.4:c.8456del:p.Pro2819LeufsTer70 | frameshift | 1/2054 | 0 | Het (*de novo*) | AD | Novel | P |
| 13193 | Profound | No | *PTPN11* | NM_002834.5:c.1528C>G:p.Gln510Glu | missense | 1/2054 | 0 | Het (*de novo*) | AD | rs397507549 | LP |
| 13197 | Profound | No | *TMPRSS3* | NM_001256317.3:c.646C>T:p.Arg216Cys | missense | 1/2054 | 1/1040 | Het | AR | rs145913750 | LP |
|  |  |  | *TMPRSS3* | NM_001256317.3:c.271C>T:p.Arg91* | nonsense | 4/2054 | 0 | Het | AR | rs199903164 | P |
| 13240 | Severe | Yes | *MYO7A* | NM_000260.4:c.1709G>A:p.Arg570Gln | missense | 1/2054 | 0 | Het | AR | rs782509064 | LP |
|  |  |  | *MYO7A* | NM_000260.4:c.6182G>A:p.Arg2061Gln | missense | 2/2054 | 0 | Het | AR | rs202175478 | LP |
| 14125 | M S | Yes | *EYA1* | NM_000503.6:c.639+2T>G | splice | 1/2054 | 0 | Het | AD | Novel | P |
| 14359 | Profound | No | *MYO15A* | NM_016239.4:c.6956+9C>G | splice | 4/2054 | 0 | Het | AR | rs1482709090 | LP |
|  |  |  | *MYO15A* | NM_016239.4:c.10250_10252del:p.Ser3417del | deletion | 5/2054 | 1/1040 | Het | AR | rs760069953 | P |
| 14722 | Profound | No | *CHD7* | NM_017780.4:c.5912del:p.Glu1971Glyfs*18 | frameshift | 1/2054 | 0 | Het (*de novo*) | AD | Novel | P |

M S: Moderately Severe; Zygosity: determined on basis of segregation analysis of the parents’ DNA sample.

Table S6. Uncertainly diagnosed patients identified in this study

| Patient | Severity  of HL | Family history | Gene | Variant | Functional  consequence | Allele frequency of patient group | Allele frequency of control group | Zygosity | Inheritance  pattern | Reference | ACMG  classification | |
| --- | --- | --- | --- | --- | --- | --- | --- | --- | --- | --- | --- | --- |
| 8170 | Profound | No | *MSRB3* | NM_198080.4:c.427G>A:p.Gly143Arg | missense | 2/2054 | 0 | Hom | AR | Novel | | VUS |
| 8635 | Profound | No | *CDH23* | NM_022124.6:c.6725T>A:p.Val2242Glu | missense | 1/2054 | 0 | Het | AR | Novel | | VUS |
|  |  |  | *CDH23* | NM_022124.6:c.8722G>C:p.Gly2908Arg | missense&splice | 1/2054 | 0 | Het | AR | Novel | | VUS |
| 8642 | Profound | No | *OTOG* | NM_001277269.2:c.746G>T:p.Gly249Val | missense | 1/2054 | 0 | Het | AR | rs1034866081 | | VUS |
|  |  |  | *OTOG* | NM_001277269.2:c.1063C>T:p.Arg355Trp | missense | 1/2054 | 0 | Het | AR | rs544751623 | | VUS |
| 8941 | Profound | No | *TBC1D24* | NM_001199107.2:c.370C>A:p.Gln124Lys | missense | 1/2054 | 0 | Het | AR | Novel | | VUS |
|  |  |  | *TBC1D24* | NM_001199107.2:c.523G>C:p.Ala175Pro | missense | 1/2054 | 0 | Het | AR | Novel | | VUS |
| 9109 | Profound | No | *MYO15A* | NM_016239.4:c.3693-2A>G | splice | 1/2054 | 0 | Het | AR | Novel | | P |
|  |  |  | *MYO15A* | NM_016239.4:c.4538C>T:p.Ala1513Val | missense | 2/2054 | 0 | Het | AR | Novel | | VUS |
| 9357 | Profound | No | *CDH23* | NM_022124.6:c.146-1G>C | splice | 1/2054 | 0 | Het | AR | Novel | | LP |
|  |  |  | *CDH23* | NM_022124.6:c.4884T>A:p.Asn1628Lys | missense | 1/2054 | 0 | Het | AR | Novel | | VUS |
| 9437 | Profound | No | *MYO15A* | NM_016239.4:c.4461C>A:p.Asn1487Lys | missense | 1/2054 | 0 | Het | AR | Novel | | VUS |
|  |  |  | *MYO15A* | NM_016239.4:c.4898T>C:p.Ile1633Thr | missense | 1/2054 | 0 | Het | AR | rs576399072 | | VUS |
|  |  |  | *MYO15A* | NM_016239.4:c.6551_6552del:p.Cys2184Serfs*34 | frameshift | 1/2054 | 0 | Het | AR | Novel | | LP |
| 9581 | Profound | No | *MYO7A* | NM_000260.4:c.6325A>C:p.Thr2109Pro | missense | 1/2054 | 0 | Het | AR | Novel | | VUS |
|  |  |  | *MYO7A* | NM_000260.4:c.6491del:p.Asn2164Thrfs*31 | frameshift | 1/2054 | 0 | Het | AR | Novel | | LP |
| 9806 | M S | No | *MYO15A* | NM_016239.4:c.4597-9C>T | splice | 2/2054 | 1/1040 | Het | AR | rs117767901 | | VUS |
|  |  |  | *MYO15A* | NM_016239.4:c.10084C>T:p.Arg3362Trp | missense | 1/2054 | 0 | Het | AR | rs759637197 | | VUS |
| 9819 | M S | Yes | *POU4F3* | NM_002700.3:c.770C>T:p.Ala257Val | missense | 1/2054 | 0 | Het | AD | Novel | | VUS |
| 9829 | Profound | No | *CDH23* | NM_001171933.1:c.1537G>T:p.Gly513* | nonsense | 1/2054 | 0 | Het | AR | Novel | | LP |
|  |  |  | *CDH23* | NM_001171933.1:c.412G>A:p.Gly138Arg | missense | 1/2054 | 0 | Het | AR | rs181197242 | | VUS |
| 9886 | Severe | No | *TMC1* | NM_138691.3:c.642+4A>C | splice | 1/2054 | 0 | Het | AR | Novel | | VUS |
|  |  |  | *TMC1* | NM_138691.3:c.1765A>G:p.Met589Val | missense | 1/2054 | 0 | Het | AR | Novel | | VUS |
| 9898 | Profound | No | *TMPRSS3* | NM_024022.4:c.371C>T:p.Ser124Leu | missense | 1/2054 | 0 | Het | AR | rs747531199 | | VUS |
|  |  |  | *TMPRSS3* | NM_024022.4:c.46C>T:p.Arg16* | nonsense | 1/2054 | 0 | Het | AR | rs976363536 | | P |
| 9936 | Profound | No | *MYO15A* | NM_016239.4:c.5507T>C:p.Leu1836Pro | missense | 1/2054 | 0 | Het | AR | rs1253612362 | | VUS |
|  |  |  | *MYO15A* | NM_016239.4:c.5835T>G:p.Tyr1945* | nonsense | 1/2054 | 0 | Het | AR | Novel | | LP |
| 10011 | Profound | No | *CDH23* | NM_022124.6:c.6050-9G>A | splice | 1/2054 | 0 | Het | AR | rs367928692 | | VUS |
|  |  |  | *CDH23* | NM_022124.6:c.9014C>T:p.Ala3005Val | missense | 1/2054 | 0 | Het | AR | rs188966938 | | VUS |
|  |  |  | *CDH23* | NM_022124.6:c.9078-7C>A | splice | 1/2054 | 0 | Het | AR | Novel | | VUS |
| 10018 | Profound | No | *LHFPL5* | NM_182548.4:c.200A>G:p.Tyr67Cys | missense | 2/2054 | 1/1040 | Het | AR | rs201738462 | | VUS |
|  |  |  | *LHFPL5* | NM_182548.4:c.462_472dup:p.Arg158Glnfs*90 | frameshift | 1/2054 | 0 | Het (*de novo*) | AR | Novel | | P |
| 10230 | Profound | No | *CDH23* | NM_022124.6:c.6368G>A:p.Gly2123Glu | missense | 1/2054 | 0 | Het | AR | Novel | | VUS |
|  |  |  | *CDH23* | NM_022124.6:c.4747G>A:p.Gly1583Ser | missense | 2/2054 | 0 | Het | AR | rs775013186 | | VUS |
| 10368 | Profound | No | *MYO15A* | NM_016239.4:c.4482+2T>C | splice | 1/2054 | 0 | Het | AR | Novel | | LP |
|  |  |  | *MYO15A* | NM_016239.4:c.5683G>A:p.Glu1895Lys | missense | 1/2054 | 0 | Het | AR | Novel | | VUS |
| 10370 | Profound | No | *MYO15A* | NM_016239.4:c.2266_2272dup:p.Pro758Argfs*224 | frameshift | 1/2054 | 0 | Het | AR | Novel | | P |
|  |  |  | *MYO15A* | NM_016239.4:c.3866C>T:p.Pro1289Leu | missense & splice | 1/2054 | 0 | Het | AR | rs192483691 | | VUS |
| 10400 | Profound | No | *CDH23* | NM_022124.6:c.3371T>G:p.Leu1124Arg | missense | 1/2054 | 0 | Het | AR | Novel | | VUS |
|  |  |  | *CDH23* | NM_022124.6:c.2833G>A:p.Gly945Ser | missense | 2/2054 | 0 | Het | AR | rs760043975 | | VUS |
| 10412 | Profound | No | *CDH23* | NM_022124.6:c.1205C>T:p.Pro402Leu | missense | 1/2054 | 0 | Het | AR | rs373168635 | | VUS |
|  |  |  | *CDH23* | NM_022124.6:c.2956del:p.Leu986Trpfs*3 | frameshift | 1/2054 | 0 | Het | AR | Novel | | LP |
| 10528 | M S | No | *MYO15A* | NM_016239.4:c.4519C>T:p.Arg1507* | nonsense | 2/2054 | 0 | Het | AR | rs549138385 | | P |
|  |  |  | *MYO15A* | NM_016239.4:c.9820T>C:p.Tyr3274His | missense | 1/2054 | 0 | Het | AR | Novel | | VUS |
| 10540 | Mild | Yes | *P2RX2* | NM_170683.4:c.1055T>G:p.Val352Gly | missense | 1/2054 | 0 | Het | AD | Novel | | VUS |
| 10736 | Moderate | Yes | *USH2A* | NM_206933.4:c.8559-2A>G | splice | 4/2054 | 4/1040 | Het | AR | rs397518039 | | VUS |
|  |  |  | *USH2A* | NM_206933.4:c.4645C>T:p.Arg1549* | nonsense | 1/2054 | 0 | Het | AR | rs199679165 | | LP |
| 10853 | Profound | No | *RECQL4* | NM_004260.4:c.2705C>T:p.Arg902Trp | missense | 1/2054 | 0 | Het | AR | rs761231404 | | VUS |
|  |  |  | *RECQL4* | NM_004260.4:c.520C>A:p.His174Asn | missense | 2/2054 | 0 | Het | AR | rs369382124 | | VUS |
| 10908 | Profound | No | *MYO15A* | NM_016239.4:c.6638G>A:p.Trp2213Ter | nonsense | 1/2054 | 0 | Het | AR | rs1567649945 | | LP |
|  |  |  | *MYO15A* | NM_016239.4:c.10350+6T>C | splice | 1/2054 | 0 | Het | AR | Novel | | VUS |
| 10920 | Profound | No | *HARS2* | NM_012208.4:c.1320G>T:p.Glu440Asp | missense | 1/2054 | 0 | Het | AR | Novel | | VUS |
|  |  |  | *HARS2* | NM_012208.4:c.322T>C:p.Tyr108His | missense | 1/2054 | 0 | Het | AR | Novel | | VUS |
| 10924 | Severe | Yes | *PJVK* | NM_001042702.5:c.880C>A:p.His294Asn | missense | 2/2054 | 0 | Hom | AR | Novel | | VUS |
| 10954 | Profound | No | *TMPRSS3* | NM_024022.4:c.551T>C:p.Leu184Ser | missense | 2/2054 | 0 | Het | AR | rs770046529 | | VUS |
|  |  |  | *TMPRSS3* | NM_024022.4:c.271C>T:p.Arg91* | nonsense | 4/2054 | 0 | Het | AR | rs199903164 | | P |
| 10963 | Profound | Yes | *TNC* | NM_002160.4:c.5335A>G:p.Ile1779Val | missense | 1/2054 | 0 | Het | AD | rs1463551263 | | VUS |
| 11041 | Profound | No | *TMC1* | NM_138691.3:c.589G>A:p.Gly197Arg | missense | 3/2054 | 0 | Het | AR | rs753687760 | | LP |
|  |  |  | *TMC1* | NM_138691.3:c.1811G>A:p.Arg604Gln | missense | 1/2054 | 0 | Het | AR | rs1484356573 | | VUS |
| 11044 | NP | No | *MYO15A* | NM_016239.4:c.3904del:p.Met1302Cysfs*10 | frameshift | 1/2054 | 0 | Het | AR | Novel | | LP |
|  |  |  | *MYO15A* | NM_016239.4:c.8474T>C:p.Leu2825Pro | missense | 1/2054 | 0 | Het | AR | Novel | | VUS |
| 11592 | Profound | No | *MYO15A* | NM_016239.4:c.5095T>G:p.Phe1699Val | missense | 1/2054 | 0 | Het | AR | Novel | | VUS |
|  |  |  | *MYO15A* | NM_016239.4:c.10250_10252del:p.Ser3417del | deletion | 5/2054 | 1/1040 | Het | AR | rs760069953 | | P |
| 11658 | Profound | No | *CDH23* | NM_022124.6:c.3972G>C:p.Glu1324Asp | missense | 1/2054 | 0 | Het | AR | Novel | | VUS |
|  |  |  | *CDH23* | NM_022124.6:c.4457A>C:p.Asp1486Ala | missense | 1/2054 | 0 | Het | AR | Novel | | VUS |
| 11902 | M S | No | *ADGRV1* | NM_032119.4:c.6514T>C:p.Ser2172Pro | missense | 1/2054 | 0 | Het | AR | Novel | | VUS |
|  |  |  | *ADGRV1* | NM_032119.4:c.4752+2T>G | splice | 1/2054 | 0 | Het | AR | rs774386059 | | LP |
| 12087 | Profound | No | *LOXHD1* | NM_144612.7:c.4212+1G>A | splice | 2/2054 | 0 | Het | AR | rs889110926 | | P |
|  |  |  | *LOXHD1* | NM_144612.7:c.4181T>A:p.Val1394Glu | missense | 1/2054 | 0 | Het | AR | rs1356532043 | | VUS |
| 12137 | Profound | No | *CDH23* | NM_022124.6:c.6606C>A:p.Asp2202Glu | missense | 1/2054 | 1/1040 | Het | AR | rs756669421 | | VUS |
|  |  |  | *CDH23* | NM_022124.6:c.5992G>A:p.Val1998Met | missense | 1/2054 | 0 | Het | AR | Novel | | VUS |
| 12192 | Profound | No | *LARS2* | NM_015340.4:c.1988G>A:p.Arg663Gln | missense | 1/2054 | 0 | Het | AR | rs201126565 | | VUS |
|  |  |  | *LARS2* | NM_015340.4:c.1795A>T:p.Ile599Phe | missense | 1/2054 | 0 | Het | AR | rs1336748553 | | VUS |
| 12221 | Profound | No | *SLC26A4* | NM_000441.2:c.919-2A>G | splice | 302/2054 | 12/1040 | Het | AR | rs111033313 | | P |
|  |  |  | *SLC26A4* | NM_000441.2:c.614G>T:p.Gly205Val | missense | 1/2054 | 0 | Het | AR | Novel | | VUS |
| 12242 | Profound | No | *PCDH15* | NM_001142763.2:c.332A>T:p.Asp111Val | missense | 1/2054 | 0 | Het | AR | Novel | | VUS |
|  |  |  | *PCDH15* | NM_001142763.2:c.769_770insAA:p.Thr257LysfsTer39 | frameshift | 1/2054 | 0 | Het | AR | Novel | | LP |
| 12253 | Profound | No | *MYO15A* | NM_016239.4:c.4538C>T:p.Ala1513Val | missense | 2/2054 | 0 | Het | AR | Novel | | VUS |
|  |  |  | *MYO15A* | NM_016239.4:c.5597T>C:p.Leu1866Pro | missense | 1/2054 | 0 | Het | AR | rs1240823956 | | VUS |
| 12513 | M S | No | *OTOA* | NM_144672.4:c.1765del:p.Gln589ArgfsTer55 | frameshift | 1/2054 | 0 | Het | AR | rs775776282 | | LP |
|  |  |  | *OTOA* | NM_144672.4:c.774A>C:p.Leu258Phe | missense | 1/2054 | 0 | Het | AR | Novel | | VUS |
| 12891 | Profound | Yes | *MYO7A* | NM_000260.4:c.1679A>G:p.Tyr560Cys | missense | 2/2054 | 0 | Het | AR | Novel | | VUS |
|  |  |  | *MYO7A* | NM_000260.4:c.5581C>T:p.Arg1861* | nonsense | 1/2054 | 0 | Het | AR | rs878864531 | | P |
| 12980 | Profound | Yes | *GJB2* | NM_004004.6:c.235del:p.Leu79Cysfs*3 | frameshift | 278/2054 | 10/1040 | Het | AR | rs80338943 | | P |
|  |  |  | *GJB2* | NM_004004.6:c.446C>A:p.Ala149Asp | missense | 1/2054 | 0 | Het | AR | Novel | | VUS |
| 13070 | Profound | No | *CDH23* | NM_022124.6:c.8782T>G:p.Phe2928Val | missense | 1/2054 | 0 | Het | AR | Novel | | VUS |
|  |  |  | *CDH23* | NM_022124.6:c.8560G>T:p.Gly2854Trp | missense&splice | 1/2054 | 0 | Het | AR | Novel | | VUS |
| 13839 | Moderate | Yes | *MYH9* | NM_002473.6:c.5184G>T:p.Glu1728Asp | missense | 1/2054 | 0 | Het | AD | Novel | | VUS |
| 14293 | Profound | No | *MYO15A* | NM_016239.4:c.9482A>C:p.Gln3161Pro | missense | 1/2054 | 0 | Het | AR | Novel | | VUS |
|  |  |  | *MYO15A* | NM_016239.4:c.4039-2A>G | splice | 1/2054 | 0 | Het | AR | rs200260574 | | LP |
| 14707 | Profound | No | *MYO15A* | NM_016239.4:c.3524dup:p.Ser1176ValfsTer14 | frameshift | 3/2054 | 0 | Het | AR | rs766187994 | | P |
|  |  |  | *MYO15A* | NM_016239.4:c.4195A>T:p.Ile1399Phe | missense | 1/2054 | 0 | Het | AR | Novel | | VUS |

Table S7. The clinical phenotype information of diagnosed and undiagnosed patients.

|  | Onset/awareness age | | | | |  |  |  |  |
| --- | --- | --- | --- | --- | --- | --- | --- | --- | --- |
|  | ≤5 | >5 | NP | Total |  |  |  |  |  |
| Diagnosed | 546 | 32 | 10 | 588 |  |  |  |  |  |
| Undiagnosed | 326 | 41 | 3 | 370 |  |  |  |  |  |
| Diagnostic rate | 62.61% | 43.84% |  |  |  |  |  |  |  |
|  | Family history | | | |  |  |  |  |  |
|  | Yes | No | NP | Total |  |  |  |  |  |
| Diagnosed | 82 | 504 | 2 | 588 |  |  |  |  |  |
| Undiagnosed | 29 | 335 | 6 | 370 |  |  |  |  |  |
| Diagnostic rate | 73.87% | 60.07% |  |  |  |  |  |  |  |
|  | Syndrome |  |  |  |  |  |  |  |  |
|  | Syndromic | Nonsyndromic | NP | Total |  |  |  |  |  |
| Diagnosed | 265 | 284 | 39 | 588 |  |  |  |  |  |
| Undiagnosed | 32 | 338 | 0 | 370 |  |  |  |  |  |
| Diagnostic rate | 89.23% | 45.66% |  |  |  |  |  |  |  |
|  | Severity | | | | | | |  |  |
|  | Mild | Moderate | M S | Severe | Profound | NP | Total |  |  |
| Diagnosed | 5 | 37 | 99 | 130 | 297 | 20 | 588 |  |  |
| Undiagnosed | 12 | 35 | 48 | 56 | 208 | 11 | 370 |  |  |
| Diagnostic rate | 29.41% | 51.39% | 67.35% | 69.89% | 58.81% |  |  |  |  |
|  | Nationality | | |  |  |  |  |  |  |
|  | Han | Minority | NP | Total |  |  |  |  |  |
| Diagnosed | 562 | 24 | 2 | 588 |  |  |  |  |  |
| Undiagnosed | 347 | 23 | 0 | 370 |  |  |  |  |  |
| Diagnostic rate | 61.83% | 51.06% |  |  |  |  |  |  |  |
|  | Geographical location | | | | | | | | |
|  | Northeast | North | Central | East | South | Northwest | Southwest | NP | Total |
| Diagnosed | 36 | 178 | 95 | 232 | 8 | 24 | 13 | 2 | 588 |
| Undiagnosed | 26 | 135 | 43 | 135 | 5 | 13 | 11 | 2 | 370 |
| Diagnostic rate | 58.06% | 56.87% | 68.84% | 63.22% | 61.54% | 64.86% | 54.17% |  |  |
|  | Gender | | |  |  |  |  |  |  |
|  | Male | Female | Total |  |  |  |  |  |  |
| Diagnosed | 305 | 283 | 588 |  |  |  |  |  |  |
| Undiagnosed | 209 | 161 | 370 |  |  |  |  |  |  |
| Diagnostic rate | 59.34% | 63.74% |  |  |  |  |  |  |  |
|  | Symmetry |  |  |  |  |  |  |  |  |
|  | Symmetric | Asymmetric | NP | Total |  |  |  |  |  |
| Diagnosed | 425 | 116 | 47 | 588 |  |  |  |  |  |
| Undiagnosed | 293 | 47 | 30 | 370 |  |  |  |  |  |
| Diagnostic rate | 59.19% | 71.17% |  |  |  |  |  |  |  |

Table S8. The information of 28 undiagnosed patients with inner malformation based on CT imaging

| Patient | Gender | Severity | Inner ear (Right) | Inner ear (Left) |
| --- | --- | --- | --- | --- |
| 8169 | F | Severe | CH-Ⅲ&CA abnormality&SCCs hypoplasia | Same^i^ |
| 8695 | M | Profound | IP-I&EVA | ND^j^ |
| 8954 | F | Profound | CC | Same |
| 8956 | F | Profound | Cochleare aplasia | IP-I |
| 9201 | F | Profound | IP-I | Same |
| 9351 | F | Profound | CH-Ⅳ | Same |
| 9412 | F | Moderate | CH-Ⅳ | Same |
| 9929 | M | Profound | Cochleare aplasia | CC |
| 9948 | M | Profound | CA abnormality&Narrow IAC | Same |
| 10002 | M | Profound | IP-I | Same |
| 10062 | M | Profound | IP-I | CC |
| 10076 | F | Profound | CA abnormality&Narrow IAC | Same |
| 10427 | M | Severe | CH-Ⅱ | Cochleare aplasia |
| 10429 | M | Profound | CA abnormality&Narrow IAC&EV&SCCs hypoplasia | Same |
| 10469 | F | Profound | Cochleare aplasia | Same |
| 10547 | F | Profound | CC | Same |
| 10575 | M | NP | CH-Ⅲ | ND |
| 10596 | M | Profound | IP-I&EVA | Same |
| 10679 | F | Moderate | CA abnormality&Narrow IAC&EV&SCCs hypoplasia | Same |
| 10940 | M | Profound | Cochleare aplasia | Same |
| 11417 | F | Profound | CH-Ⅱ | Same |
| 11459 | F | Moderate | CH-Ⅳ | Same |
| 11726 | M | Profound | CH-Ⅳ | Same |
| 11792 | F | Profound | Cochleare aplasia | Same |
| 12863 | M | Profound | Rudimentary otocyst | CC |
| 13054 | F | Profound | CA abnormality&Narrow IAC | Same |
| 13603 | M | Profound | CC | Same |
| 14081 | F | Profound | CH-Ⅱ | Same |

CH: cochleare hypoplasia; CC: common cavity; IP: incomplete partition of cochlear; EV: enlarged vestibular; EVA: enlarged vestibular aqueduct; CA: cochlear aperture; IAC: internal auditory canal; SCCs: semicircular canals; Same: bilateral ears with the same malformation of inner ear; ND: not determined because of cochlear implantation.


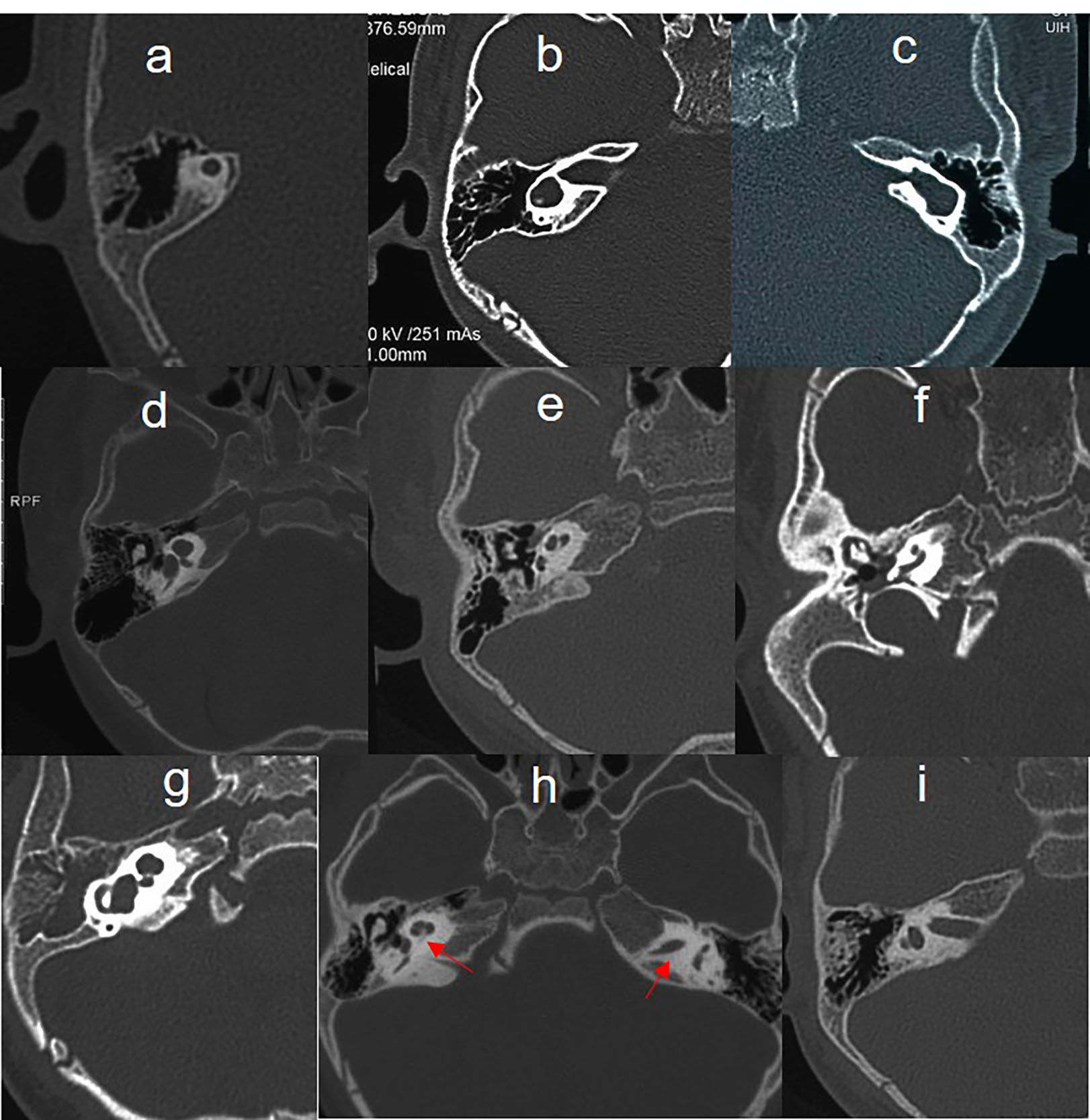


Figure S1. The inner ear malformations identified in this study. a: Rudimentary otocyst; b: Cochleare aplasia; c: common cavity; d: cochleare hypoplasia II type/CH-II; e: CH-III; f: CH-IV; g; cochlear incomplete partition type I (IP-I); h: cochlear aperture abnormality & Narrow internal auditory canal; i: semicircular canals hypoplasia.
